# Supplementary material for: The microprotein Minion controls cell fusion and muscle formation
Source: Nat Commun. 2017 Jun 1;8:15664. doi: 10.1038/ncomms15664 (PMC5461507; doi:10.1038/ncomms15664)
Supplement: Supplementary Information — Supplementary figures, supplementary tables and supplementary references. [file ncomms15664-s1.pdf]

a

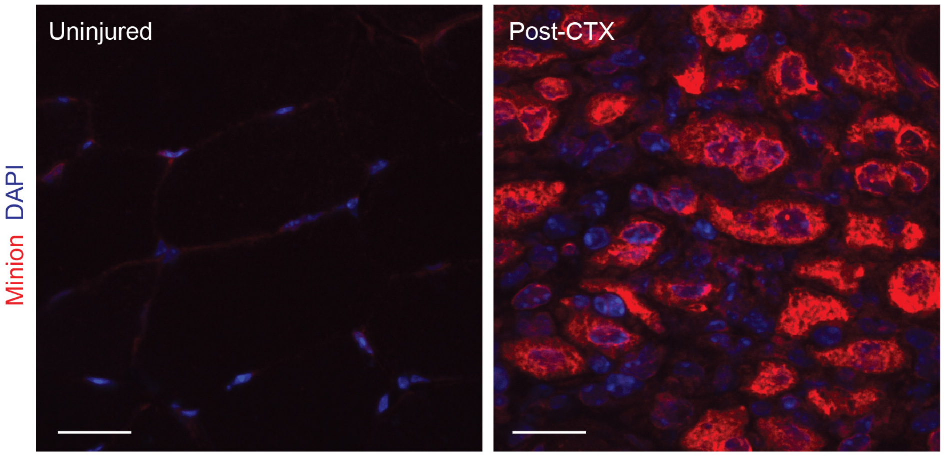

b

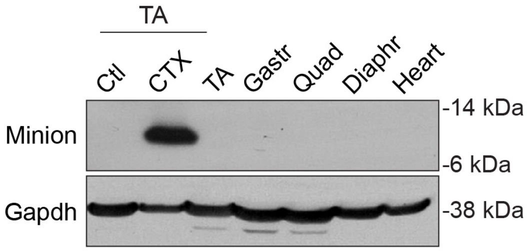

c

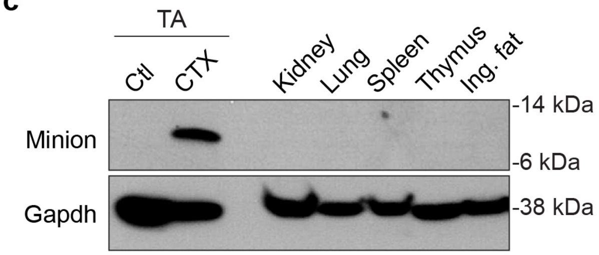

d

Somite stage	4	5	6	7	8	9	10	11	12	13	14	15	16	17
MyoD1	0.1	0.1	0.1		0.1				0.1					
Minion												0.1		

Somite stage	18	19	20	21	22	23	24	25	26	27	28	34	35	36
MyoD1							0.1	0.1	0.1	0.1	0.2	1	1	2
Minion	0.2		0.1	0.1	0.1	0.1	0.1	0.1	0.4	0.2	0.2	0.5	0.3	0.7

e

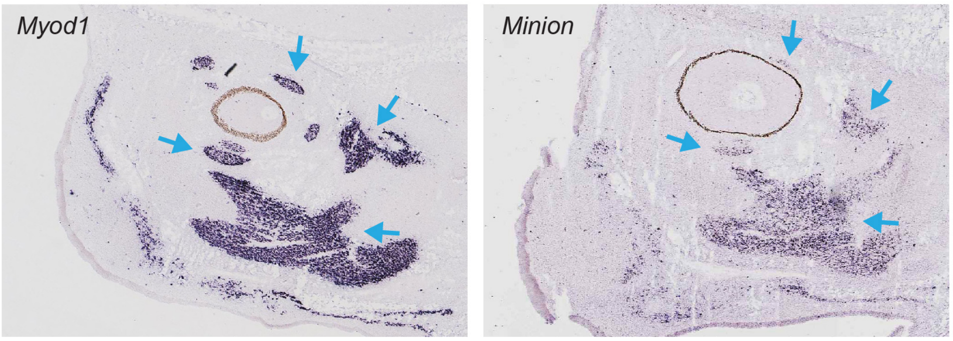

Supplementary Fig. 1. Specific expression of Minion in regenerating and developing skeletal muscle. (a) Immunofluorescence staining of transverse sections prepared from fresh frozen uninjured (left) or regenerating (right, 3 to 4 days post CTX injection) TA muscle of 10-week-old mice. Minion (red) and DAPI (blue) are shown. Scale bars: 20 µm. n=2 (5 different sections each). **(b)** Western blot analysis of various adult skeletal muscle groups and of cardiac muscle using the indicated antibodies. 6- to 8-week-old

mice were used. n=2, and 2 technical replicates each. Ctl: uninjured TA muscle; CTX: regenerating TA muscle at day 4 post CTX injection; TA: *tibialis anterior*; Gastr: *gastrocnemius*; Quad: *quadriceps femoris*; Diaphr: diaphragm. **(c)** Western blots analysis using the indicated antibodies of various adult tissues from 6-8 week old mice. n=2, and 2 technical replicates each. **(d)** Expression of mouse *Minion* during embryonic development as detected by RNA-seq (EMBL-EBI Expression Atlas; http://www.ebi.ac.uk/gxa)[1,2]. Numbers indicate RNA expression level (FPKM). **(e)** Expression of mouse *myod1* (left) and *Minion* (right) in non-somitic muscle as detected by *in situ* hybridization at E14.5 (Eurexpress; http://www.eurexpress.org/ee/intro.html)[3]. Blue arrows indicate overlapping expression in extraocular and facial muscle.

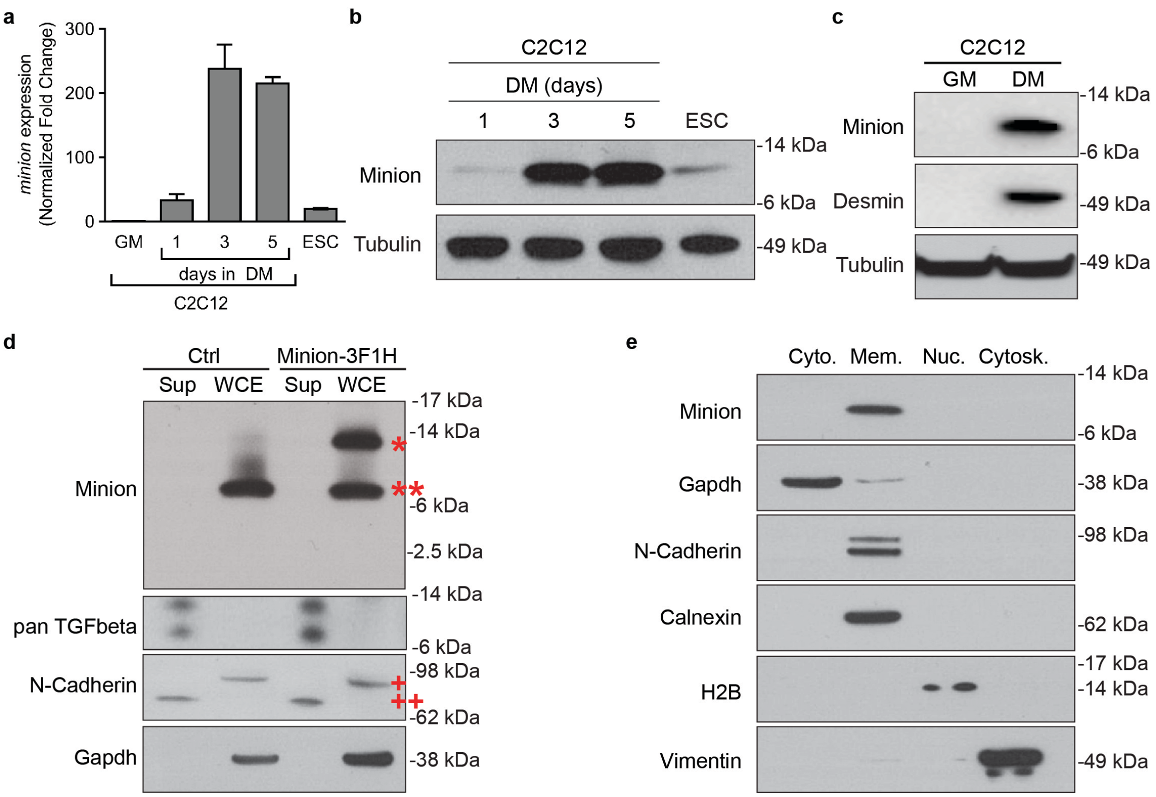

Supplementary Fig. 2. *Minion* is highly expressed in differentiating muscle cells and is membrane associated. (a) RT-qPCR quantification of *Minion* mRNA levels in C2C12 myoblasts under growth (GM) or differentiation (DM) conditions. Mouse CJ7 embryonic stem cells (ESC) served as a positive control. n=3. Each value represents mean±s.d. **(b)** Western blot analysis of differentiating C2C12 myoblasts (at day1, 3, 5 in DM), and of embryonic stem cells (ESC) using the indicated antibodies. n=2. **(c)** Western blots analysis of C2C12 myoblasts in GM or at day3 in DM using the indicated antibodies. Desmin is an intermediate filament protein high in differentiated muscle cells. n=2. **(d)** Western blot analysis of concentrated cell culture supernatant (Sup) from day 3 differentiating C2C12 myoblasts expressing control vector or C-terminally 3×FLAG-1×HA-tagged Minion. Both tagged (*) and endogenous (**) Minion are detected in whole cell extract (WCE). TGFβ and cleaved N-Cadherin (++) are positive controls, whereas Gapdh and intact N-Cadherin (+) are non-secreted negative controls. n=3. **(e)** Subcellular fractionation of C2C12 myoblasts at day 4 in DM. Western blot analysis was performed using the indicated antibodies. The four fractions examined were cytosolic (Cyto), membrane (Mem, nuclear (Nuc), and cytoskeletal (Cytosk). The membrane fraction contains both plasma membrane as well as intracellular membranes (ER, Golgi, mitochondria, endosome, lysosome, etc.). Gapdh: cytosolic fraction marker; N-Cadherin: plasma membrane marker; Calnexin: ER membrane marker; Histone H2B: nuclear fraction marker; Vimentin: cytoskeletal fraction marker. n=3.

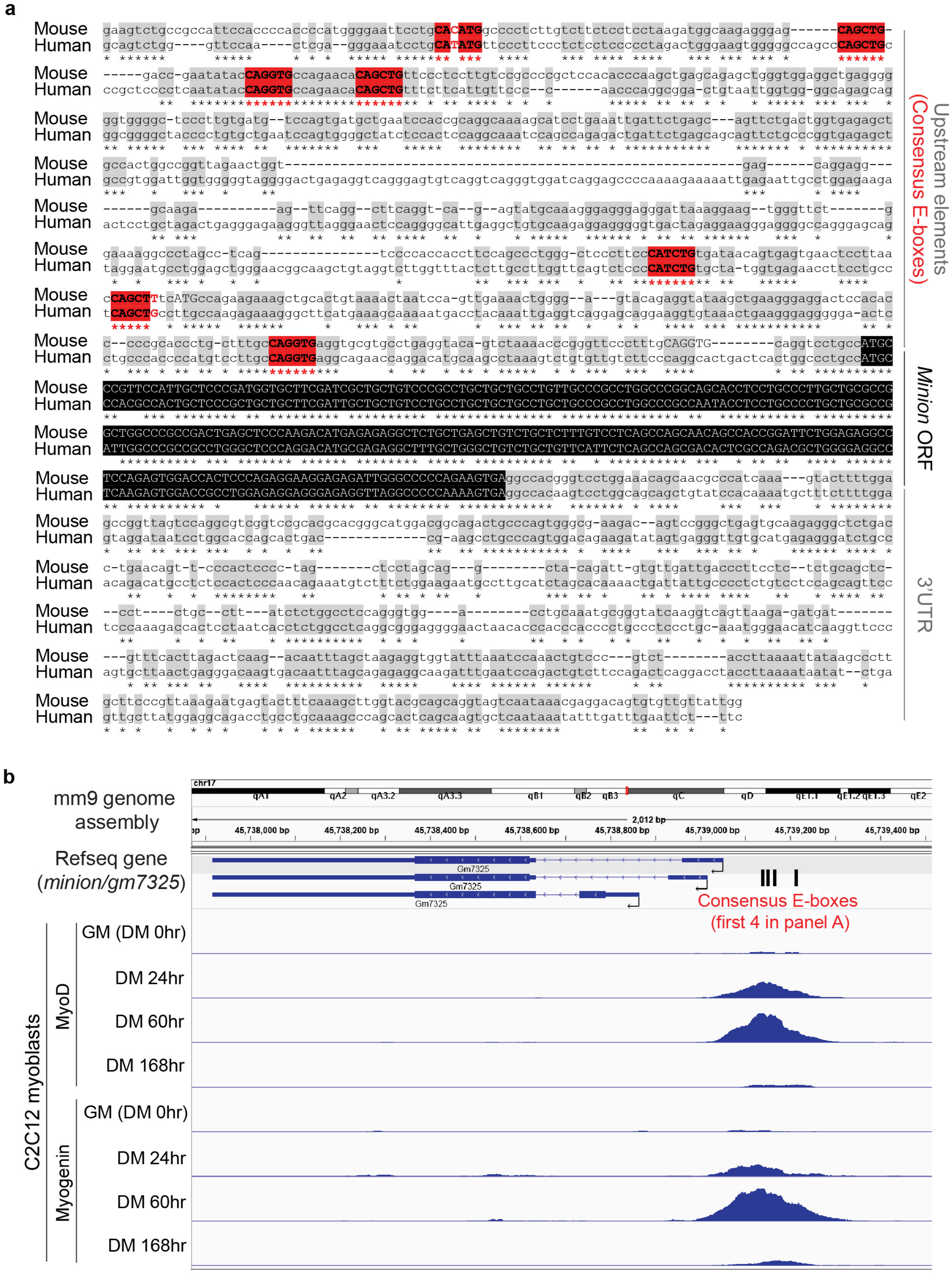

Supplementary Fig. 3. ***Minion*** **promoter structure is consistent with regulation by MyoD and Myogenin. (a)** Sequence alignment of the genomic regions surrounding mouse *gm7325/Minion* gene and its human ortholog *RP1-302G2.5* (Ensembl gene name, a.k.a. NCBI gene name *LOC101929726*). The *Minion* ORF is highlighted in black. Seven

conserved E-box motifs (CANNTG and CANNTT) within the promoter and 5'UTR region are highlighted in red. **(b)** ENCODE MyoD and Myogenin ChIP-seq data from C2C12 myoblasts under growth conditions (GM) and at three time points under differentiation conditions (DM) were examined surrounding the *Minion* genomic locus. Black bars indicate the first four conserved E-box motifs within the promoter region in (a).

a

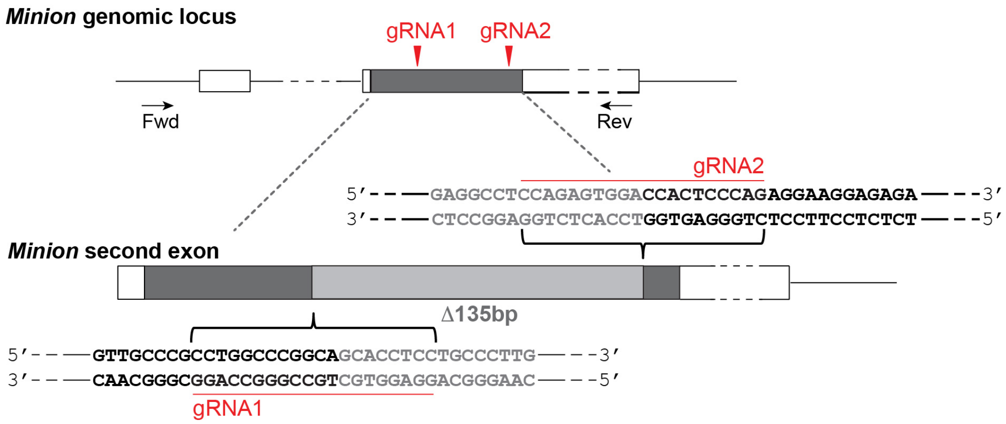

b

```
Minion -FL(84AA)   MPVPLLPMVLRSLLSRLLLPVARLARQHLLPLLRRLARRLSSQDMREALLSCLLFVLSQQQPPDSGEASRVDHSQRKERLGPQK
Truncated-39AA     MPVPLLPMVLRSLLSRLLLPVARLARH---------------------------------------------HSQRKERLGPQK
```

c

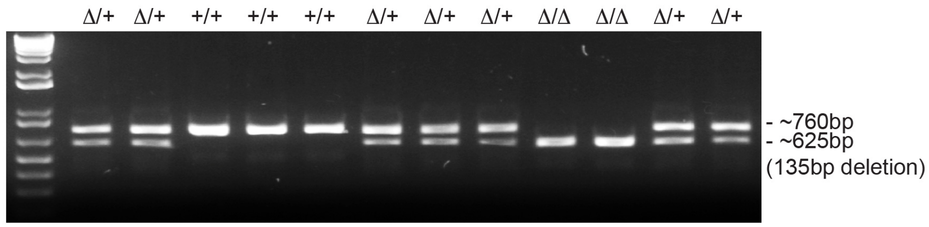

d

Het x Het	+/+	Δ/+	Δ/Δ	Total number
E18.5-E19.5 embryos	19 (23%)	47 (57%)	17 (20%)	83
Adult mice	51 (31%)	111 (69%)	0	162

Supplementary Fig. 4. Generation of *Minion*-deficient mice by CRISPR/Cas9 gene editing. (a) Detailed schematic of Fig.2a for generation of *Minion*-deficient animals by CRISPR/Cas9 mediated editing. A 135bp in-frame deletion (marked in light gray) within the *gm7325/Minion* ORF (gray) was induced using a double sgRNA approach. The genomic target sequences of the gRNAs are indicated with a red line. Fwd: forward PCR genotyping primer; Rev: reverse PCR genotyping primer. **(b)** Protein sequence alignment of the 84 aa full-length mouse Minion and the predicted 39 aa truncated form ($Minion^{\Delta}$). The truncated form is predicted to contain only the N-terminal 26 aa and C-terminal 12 aa of the original microprotein. **(c)** Agarose gel picture of typical genotyping PCR results using E18.5 embryos from $Minion^{\Delta/+}$ × $Minion^{\Delta/+}$ crosses (Het×Het). +/+: wild type ($Minion^{+/+}$); Δ/+: heterozygote ($Minion^{\Delta/+}$); Δ/Δ: *Minion* knockout homozygote ($Minion^{\Delta/\Delta}$). n=12 (83 E18.5-E19.5 embryos) **(d)** Summary of genotyping results of both late-stage embryos and adult mice from $Minion^{\Delta/+}$ × $Minion^{\Delta/+}$ crosses.

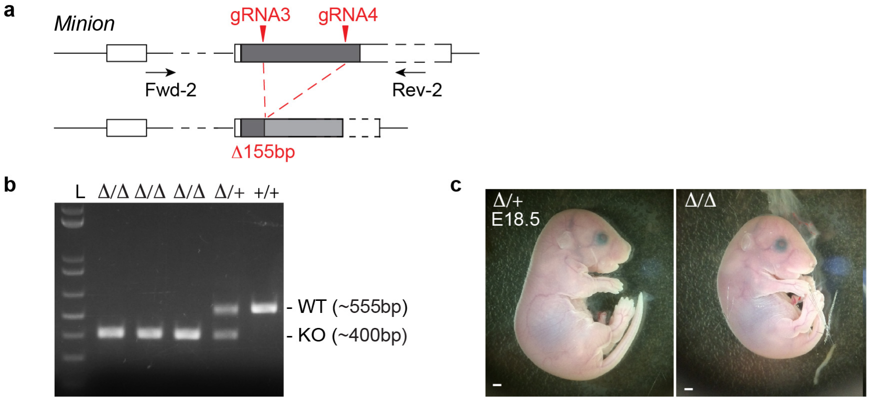

d

Minion-FL (84AA) **MPVPLLPMVLRSLLSRLL**LPVARLARQHLLPLLRRLARRLSSQDMREALLSCLLFVLSQQQPPDSGEASRVDHSQRKERLGPQK

Minion-155KO (74AA) **MPVPLLPMVLRSLLSRLL**SGPLPEEGEIGPPEVRPRVLETATPIKVLLEPVSPGVGPHARAWTADCPVGEDSPG

e

Het x Het	+/+	Δ/+	Δ/Δ	Total number
E16.5-E18.5 embryos	5	12	**9**	26
Adult mice	18	26	**0**	44

Supplementary Fig. 5. Generation of a second *Minion* knockout allele by CRISPR/Cas9 gene editing. (a) Strategy for CRISPR/Cas9 mutagenesis of the *gm7325/Minion* locus using a dual sgRNA approach with two different sgRNAs (gRNA3 and gRNA4). Gray box, *Minion* ORF; white box, non-coding exons; gRNA, single guide RNA; light gray box, new coding region after the 155bp deletion; Fwd-2 and Rev-2, forward and reverse genotyping primers to detect the 155bp deletion. **(b)** Agarose gel picture of typical genotyping PCR results using E16.5 to E18.5 embryos from $Minion^{\Delta/+}$ × $Minion^{\Delta/+}$ crosses. +/+: wild type ($Minion^{+/+}$); Δ/+: heterozygote ($Minion^{\Delta/+}$); Δ/Δ: *Minion* knockout homozygote ($Minion^{\Delta/\Delta}$). n=5 (26 E16.5-E18.5 embryos total). **(c)** Photographs of unskinned $Minion^{\Delta/+}$ and $Minion^{\Delta/\Delta}$ E18.5 embryos. n=3. Scale bars: 1mm. **(d)** Protein sequence alignment of the 84 aa full-length mouse Minion (Minion-FL) and the predicted 74 aa truncated form (Minion-155KO). The truncated form is predicted to contain only the N-terminal 18 aa of the original protein (in bold). **(e)** Summary of genotyping results of both late-stage embryos (E16.5 to E18.5) and adult mice from $Minion^{\Delta/+}$ × $Minion^{\Delta/+}$ crosses.

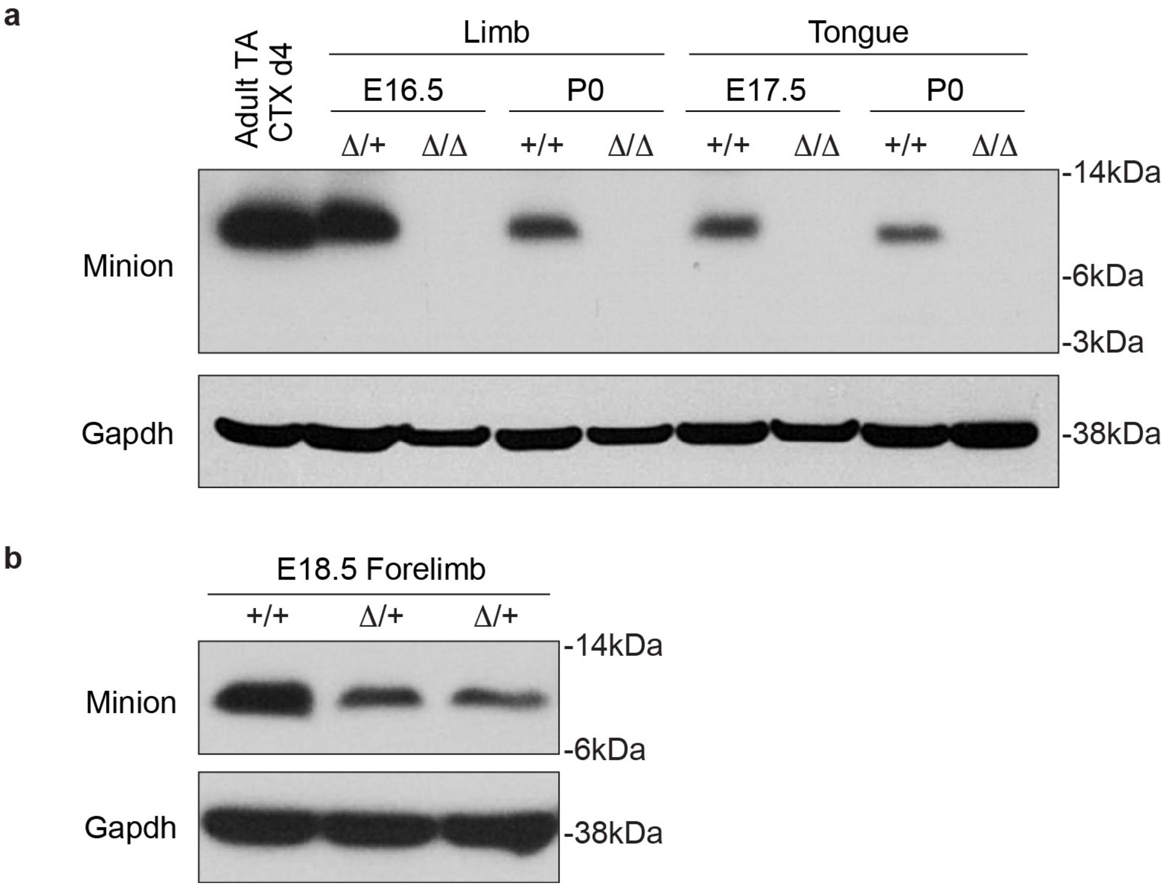

Supplementary Fig. 6. Loss of Minion protein in *Minion*-deficient animals. (a) Western blot analysis of limbs and tongues from E16.5/E17.5 embryos and P0 mice using indicated antibodies. Adult TA muscle (10 week old) at 4 days post cardiotoxin injection (CTX 4dpi) was used as a positive control. Embryos from the same litter were used for each comparison. n=3. The full-length Minion was not observed in the $Minion^{\Delta/\Delta}$ embryos, and the predicted 39 aa truncated protein was not observed using the same antibody. **(b)** Western blot analysis of forelimbs from $Minion^{+/+}$and $Minion^{\Delta/+}$ E18.5 embryos of the same litter using indicated antibodies.

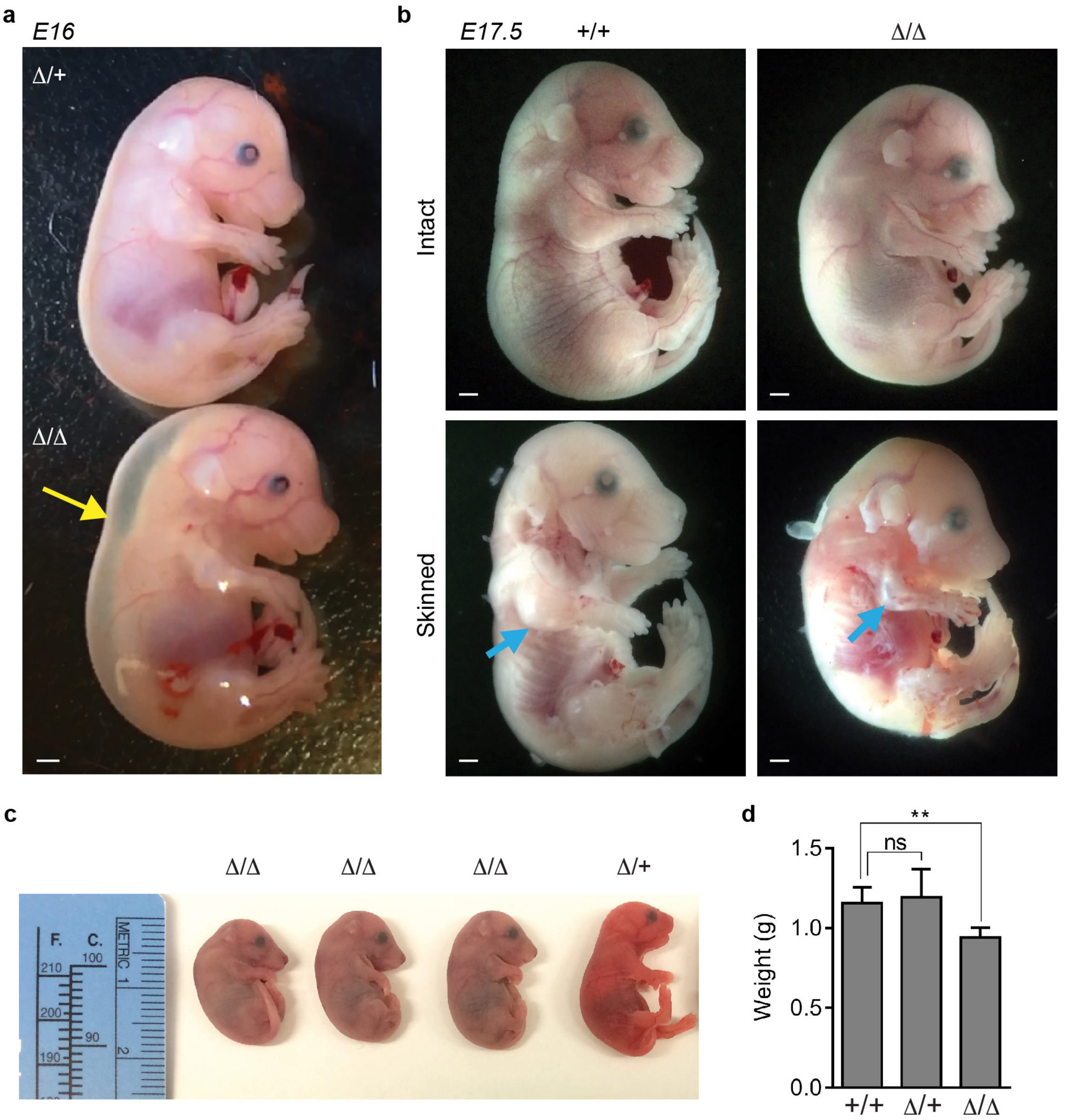

Supplementary Fig. 7. Developmental abnormalities in *Minion*-deficient animals. (a) Photos of *Minion*$^{\Delta/+}$ and *Minion*$^{\Delta/\Delta}$ E16 embryos. Yellow arrow indicates accretion of dorsal and nuchal subcutaneous edema. n=3. Scale bars: 1 mm. **(b)** Photos of *Minion*$^{+/+}$ (left) and *Minion*$^{\Delta/\Delta}$ (right) E17.5, either unskinned (top) or skinned (bottom). Cyan arrow indicates expected location of forelimb musculature. n=3. Scale bars: 1 mm. **(c)** Photos of E18.5 embryos of the indicated genotypes following air breathing after delivery by cesarean section. The *Minion*$^{\Delta/\Delta}$ embryos were atonic and exhibited an abnormal spinal curvature, and became cyanotic and died soon after delivery. n=5. **(d)** Quantification of E18.5 embryo weight after delivery by cesarean section (33 embryos total). NS: not significant; double asterisks: *P <0.001*; unpaired two-tailed Student's t-test.

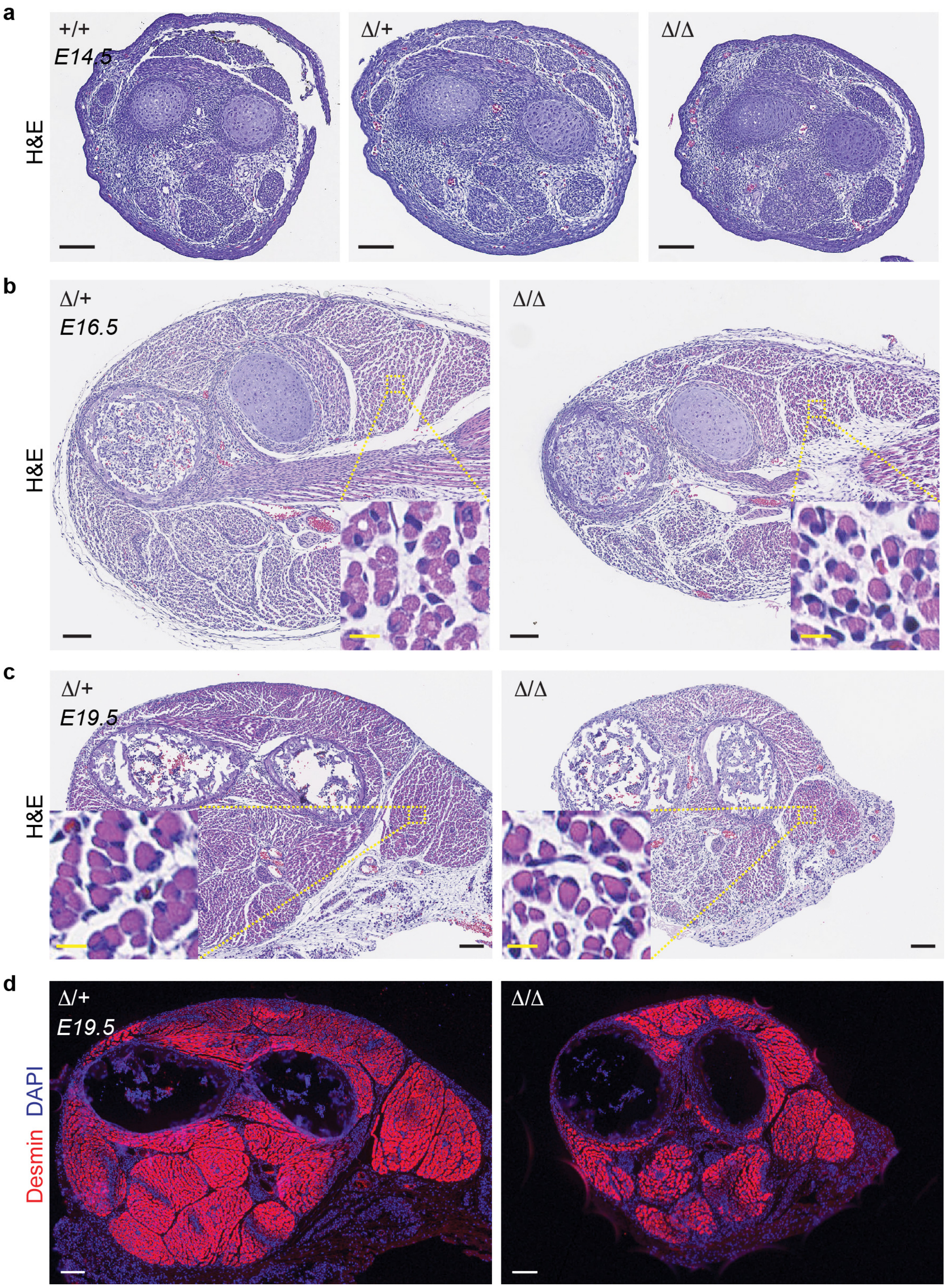

Supplementary Fig. 8. Minion deficiency affects limb muscle formation. Paraffin-embedded embryos of different stages were examined. **(a)** Histological images of hematoxylin and eosin (H&E) stained forelimb transverse sections of E14.5 embryos with indicated genotypes. No clear difference is observed in knockout animals at this

early stage. n=2. **(b)** Histological images of H&E-stained E16.5 forelimb transverse sections of indicated genotypes. Inset demonstrates magnification of the region shown in yellow box. n=2. Muscle groups in *Minion*$^{\Delta/\Delta}$ animals appear smaller than in controls. **(c)** Histological images of H&E-stained forelimb transverse sections for E19.5 embryos with indicated genotypes. Inset demonstrates magnification of the region shown in yellow box. n=3. Muscle groups in *Minion*$^{\Delta/\Delta}$ animals are smaller and more sparsely distributed than in controls. **(d)** Immunofluorescence images of forelimb transverse sections for E19.5 embryos with indicated genotypes. Desmin (red) marks all differentiating myoblasts, myotubes, and muscle fibers in the embryo. Loss of Minion does not obviously block the differentiation of skeletal muscle during development. n=3. Nuclei are labeled by DAPI (blue). Black and white scale bars: 100 µm; yellow scale bars: 10 µm.

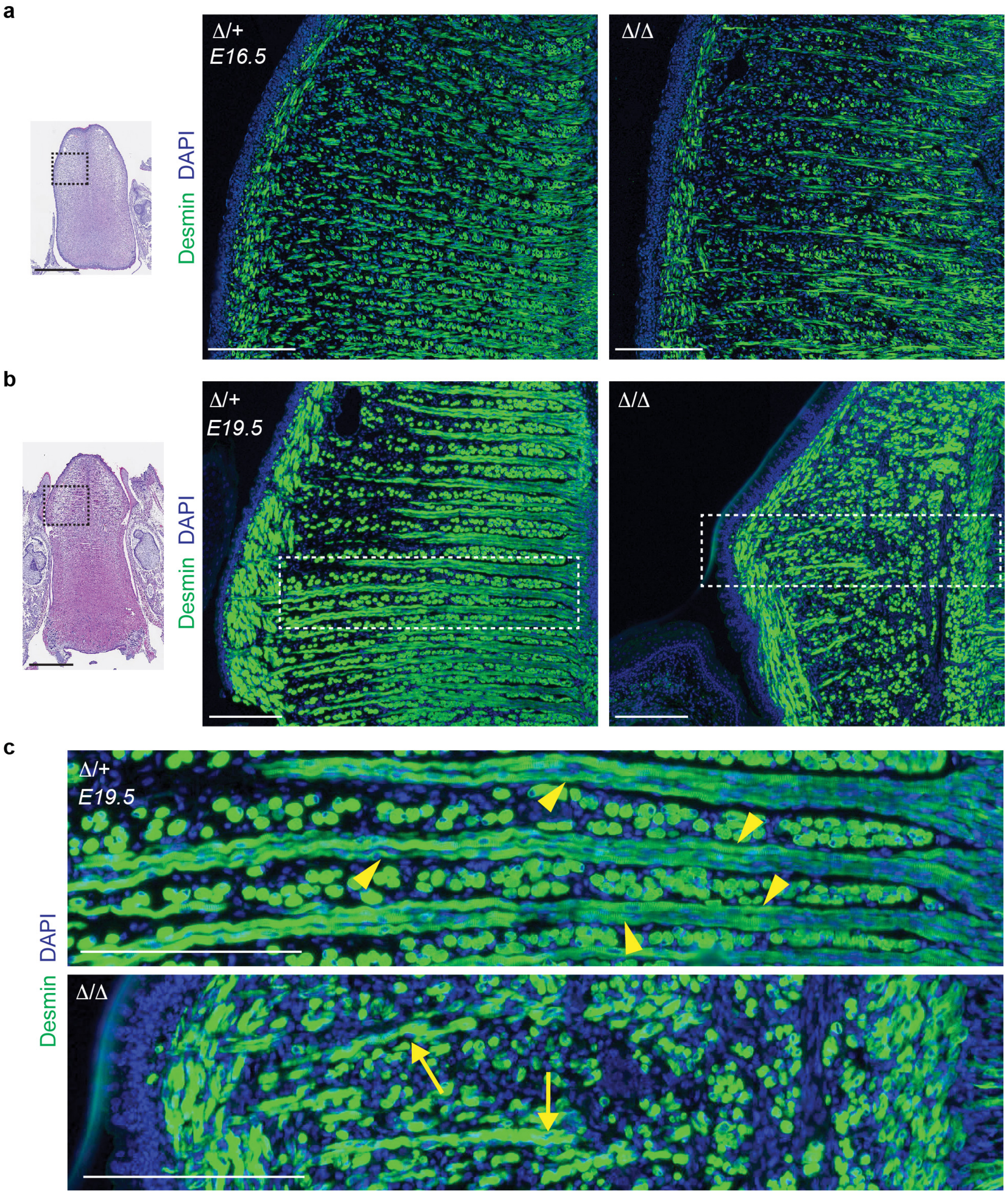

Supplementary Fig. 9. Loss of Minion *in vivo* blocks muscle cell fusion but does not affect muscle progenitor differentiation . Paraffin-embedded embryonic tongues of different stages were used to examine both longitudinal and transverse myofibers. **(a)** Immunofluorescence images of E16.5 tongue transverse sections. Black box in histological image at left demonstrates the area shown at right in fluorescence images. n=2. Desmin (green) and DAPI (blue) are shown. **(b)** Immunofluorescence images of E19.5 tongue transverse sections. Black box in histological image at left demonstrates the area shown at right in fluorescence images. n=3. White box indicates area magnified in (c). Desmin (green) and DAPI (blue) are shown. **(c)** Magnified view of white boxed area

shown in (b), demonstrating significant reduction in polynucleated myofibers in *Minion*$^{\Delta/\Delta}$ tongue. Yellow arrowheads and yellow arrows indicate fused multinuclear myofibers and unfused differentiating and elongating myoblasts respectively. Scale bars: 1 mm for H&E images; 200 μm for immunofluorescence images.

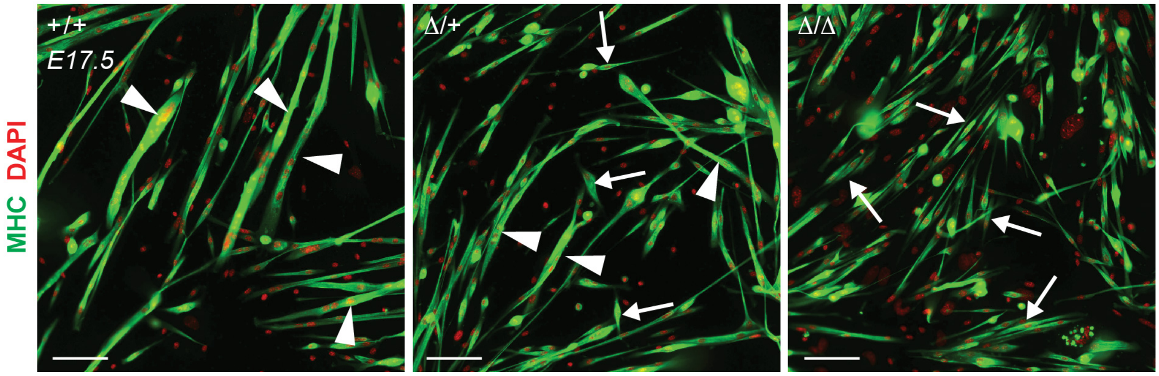

Supplementary Fig. 10. Genetic loss of Minion blocks embryonic myoblast fusion *in vitro*. Immunofluorescence images of E17.5 primary embryonic myoblasts of indicated genotypes following 3 days in differentiation medium. White arrowheads and white arrows point out the fused multinuclear myotubes and unfused differentiating elongating myoblasts. n=2 (5 technical replicates each). MHC (green) and DAPI (red) are shown. Scale bars: 100 μm.

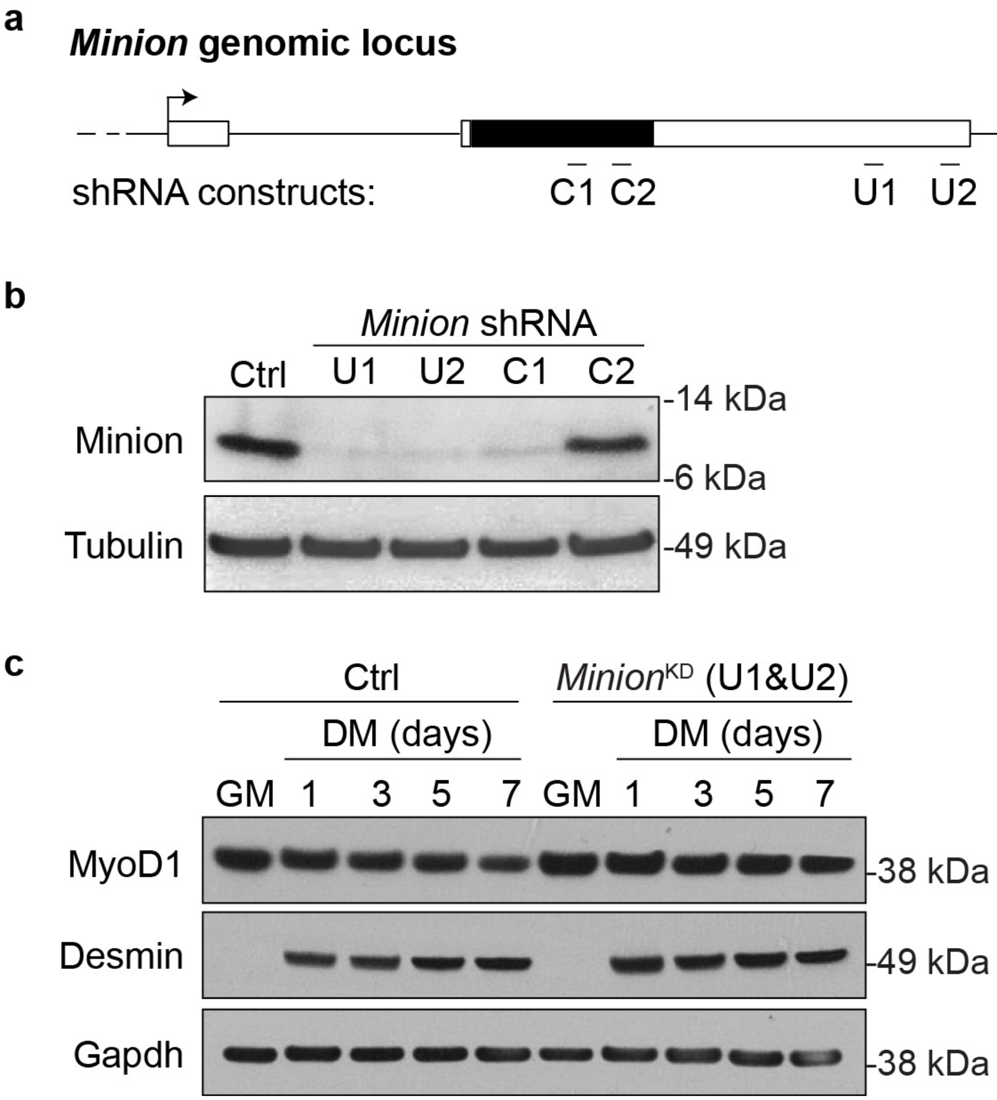

Supplementary Fig. 11. Validation of lentiviral shRNA constructs targeting mouse *Minion*. **(a)** Schematic of the mouse *Minion* genomic locus with the target regions of four shRNA constructs underlined. Solid black bar indicated the *Minion* ORF, and white bar indicated untranslated regions (UTRs). C1 and C2: shRNA constructs targeting the coding sequence; U1 and U2: shRNA constructs targeting the 3'UTR. **(b)** Western blot analysis of C2C12 myoblasts transduced with the indicated lentiviral shRNA constructs. A shRNA construct targeting the firefly (*Photinus pyralis*) *luciferase* gene was used as a negative control (Ctrl). After lentiviral infection and GFP sorting, the cells were expanded and kept in differentiation medium for 5 days. The two shRNA constructs targeting the *Minion* 3'UTR (U1/U2) were found to reduce Minion expression most efficiently and for subsequent experiments in C2C12 and primary myoblasts, cells were infected with U1 and U2 shRNA viruses and sorted by GFP after each round of infection to generate *Minion*^{KD} cells. Similarly, C2C12 cells were infected with the control virus in two rounds and sorted twice by GFP to generate the control cells (Ctrl) below. n=2. **(c)** Western blot analysis of Ctrl and *Minion*^{KD} C2C12 cells cultured in either growth medium (GM), or in differentiation medium for indicated number of days with indicated antibodies. n=3. Data are an expanded version of those shown in Fig. 5c.

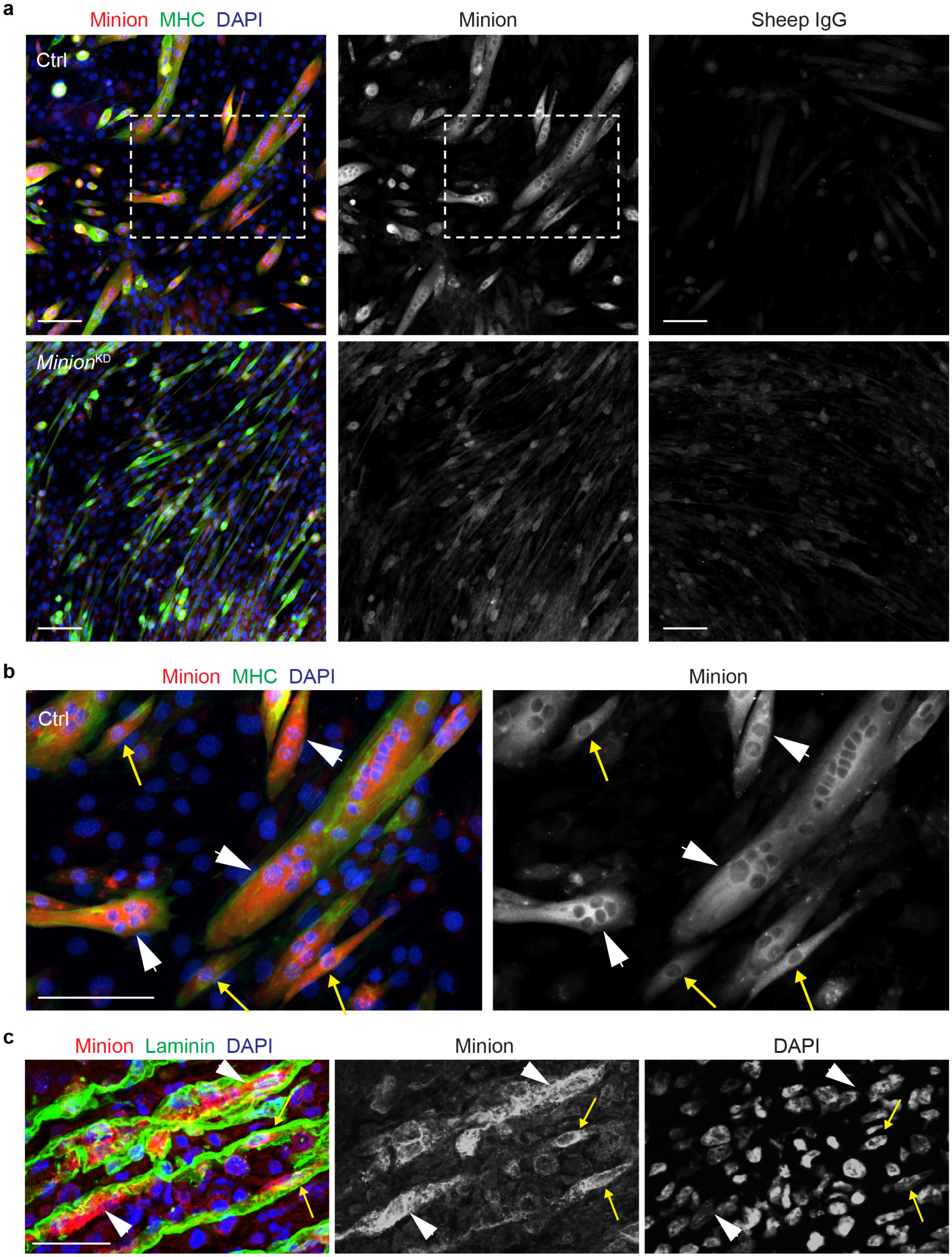

Supplementary Fig. 12. Minion is expressed in differentiating myoblasts and nascent myotubes. (a) Immunofluorescence images of Ctrl and *Minion*^{KD} C2C12 myoblasts and myotubes after 3.5 days in DM. Left: Minion (red), MHC (green) and DAPI (blue) are shown; Middle: grayscale images for Minion only; Right: grayscale

images for control IgG. Insets marked by white dotted lines are magnified in (b). n=2. **(b)** Enlarged images of the insets in (a). White arrowheads indicate the fused multinuclear myotubes and yellow arrows indicate unfused differentiating elongating myoblasts, both of which express Minion protein. **(c)** Immunofluorescence staining of longitudinal sections of regenerating adult (10 week old) TA muscle 3 days post CTX injection. Left: Minion (red), Laminin (green) and DAPI (blue); Middle: Minion only (grayscale); Right: DAPI (grayscale). White arrowheads indicate nascent myotubes and yellow arrows indicate unfused differentiating elongating myoblasts, both of which express Minion protein. n=3. Scale bars: (a, b) 100 μm and (c) 30 μm.

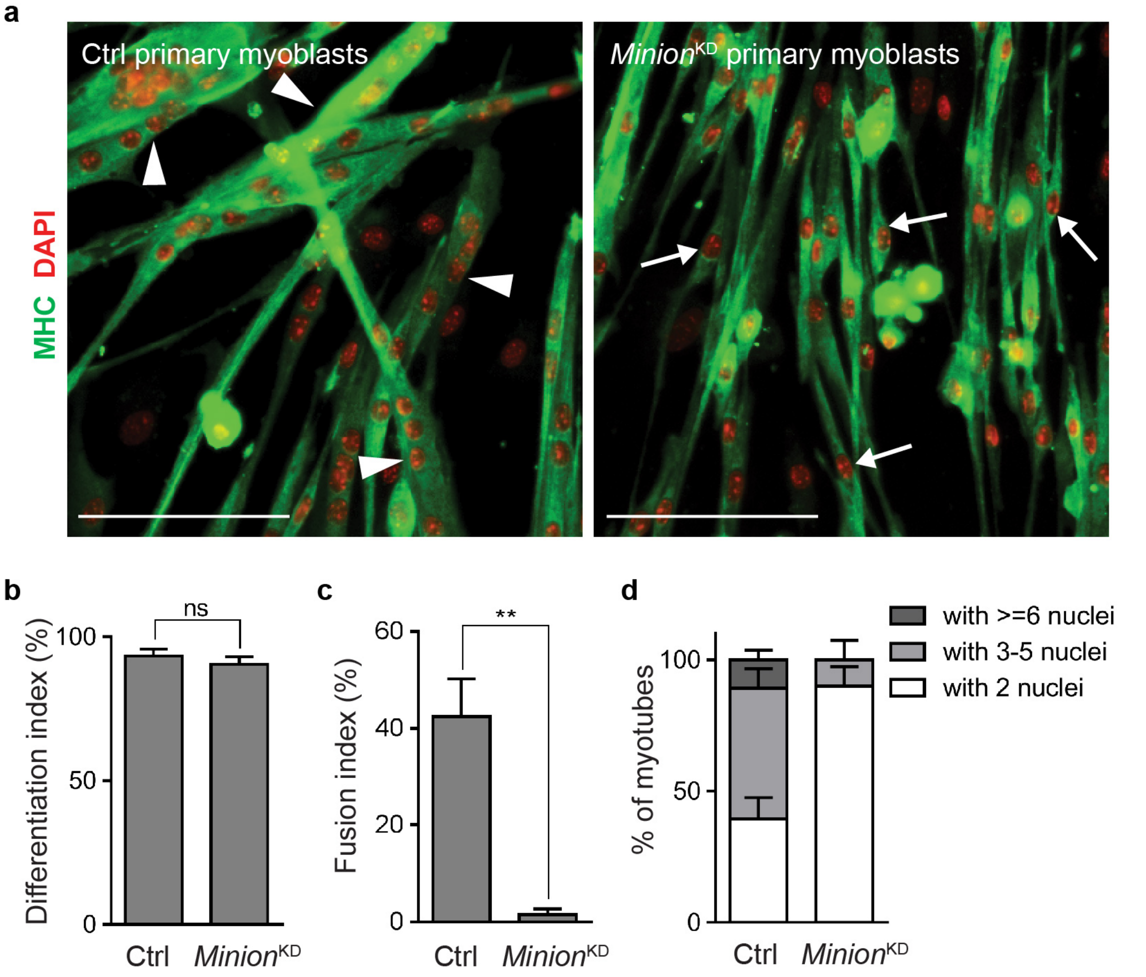

Supplementary Fig. 13. Knockdown of Minion in primary adult myoblasts blocks myoblast fusion. (a) Immunofluorescence images of Ctrl and *Minion*^{KD} primary myoblast-derived myofibers, formed after 4 days in differentiation medium. White arrowheads indicate fused multinuclear myotubes and white arrows indicate unfused differentiating elongating myoblasts. n=2 (6 technical replicates each). MHC (green) and DAPI (red) staining are shown. Scale bars: 100 µm. **(b)** Quantification of differentiation index for the experiment done in (a). Differentiation index was calculated as the percentage of nuclei within MHC^{+} cells among total nuclei in each field. NS: not significant. **(c)** Quantification of fusion index for the experiment done in (a). Fusion index was calculated as the percentage of nuclei within MHC^{+} myotubes containing ≥3 nuclei among total nuclei in each field **(d)** Quantification of the percentage of myotube numbers for the experiment done in (a). Myotubes were binned by nuclear number as indicated, and the percentage of myotubes within each subgroup was calculated. Double asterisks: P <0.001. b-d, Each value reported represents mean±s.d. n=6 (one 0.7mm×0.7mm field each), and unpaired two-tailed Student's t-test was used.

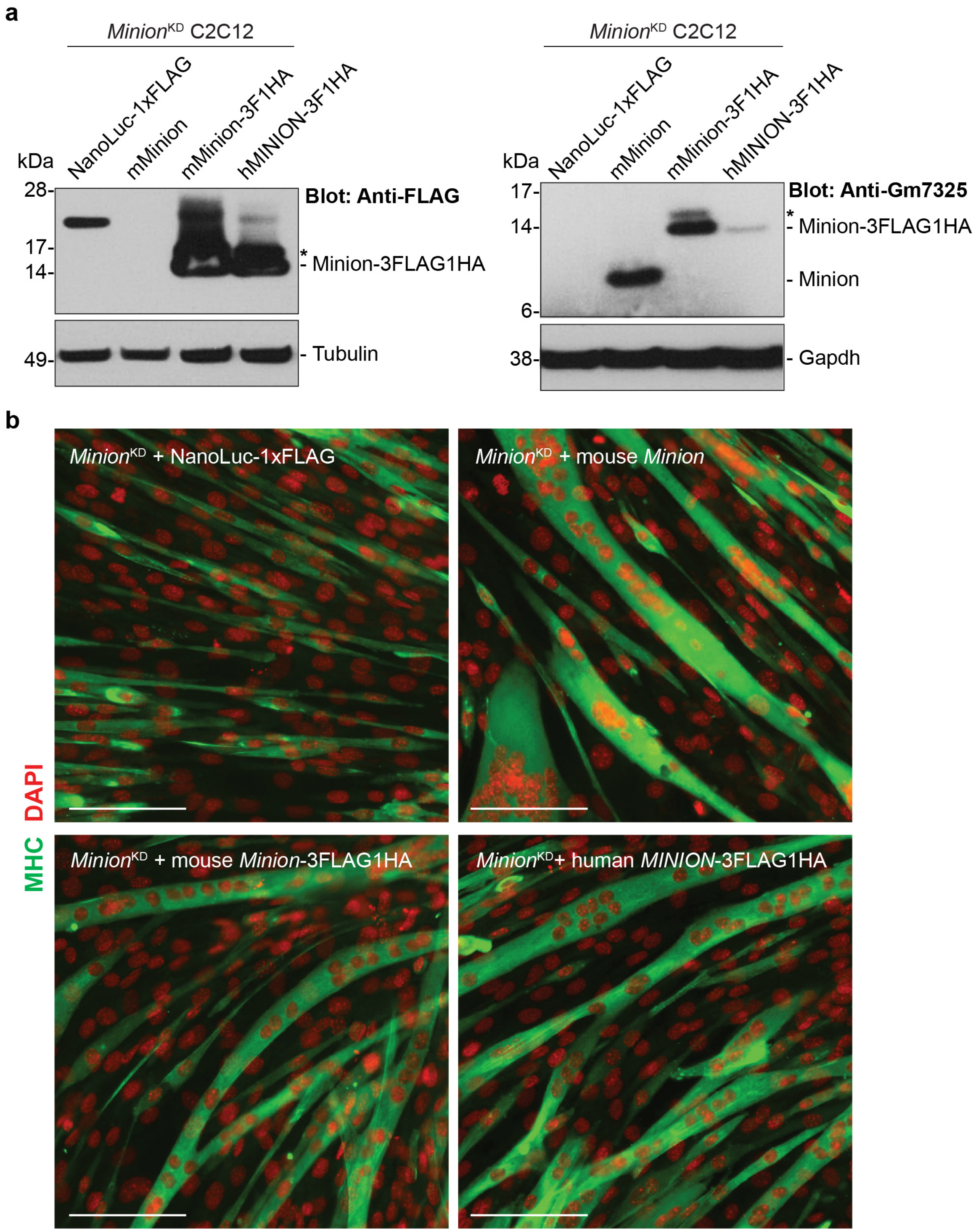

Supplementary Fig. 14. Exogenous expression of the human Minion ortholog rescues the fusion defect of *Minion*^{KD} myoblasts. (a) Western blots analysis of *Minion*^{KD} cells after retroviral expression of untagged and C-terminally tagged Minion and culture in differentiation medium for 5 days. Retroviral vectors carrying full-length mouse *Minion* CDS, C-terminally 3×FLAG-1×HA-tagged mouse *Minion* CDS and C-terminally 3×FLAG-1×HA-tagged human *MINION* ortholog CDS were used for reconstitution. A C-terminally 1×FLAG-tagged Nanoluc retroviral vector was used as

negative control. The anti-FLAG antibody recognizes three tagged proteins at the correct size, while the anti-Minion antibody not only recognizes the mouse Minion protein (tagged and untagged) but also weakly recognizes the human *MINION* ortholog. Note that the pCIGAR Gateway retroviral vectors carrying tagged human and mouse *Minion* CDS inherited an extra upstream in frame start codon, giving rise to an extra band of slightly larger size (16 aa larger, asterisk); for all other pCIGAR constructs, an extra T was added before the Kozak sequence to avoid this effect. n=2. **(b)** Immunofluorescence images of *Minion*^{KD} C2C12 cells with exogenous expression of tagged NanoLuc, untagged mouse Minion, tagged mouse Minion and tagged human *MINION* ortholog after 5 days in differentiation medium. MHC (green) and DAPI (red) staining are shown. n=3 (8 technical replicates each). Data are an expanded version of those shown in Fig. 5h. Scale bars: 100 μm.

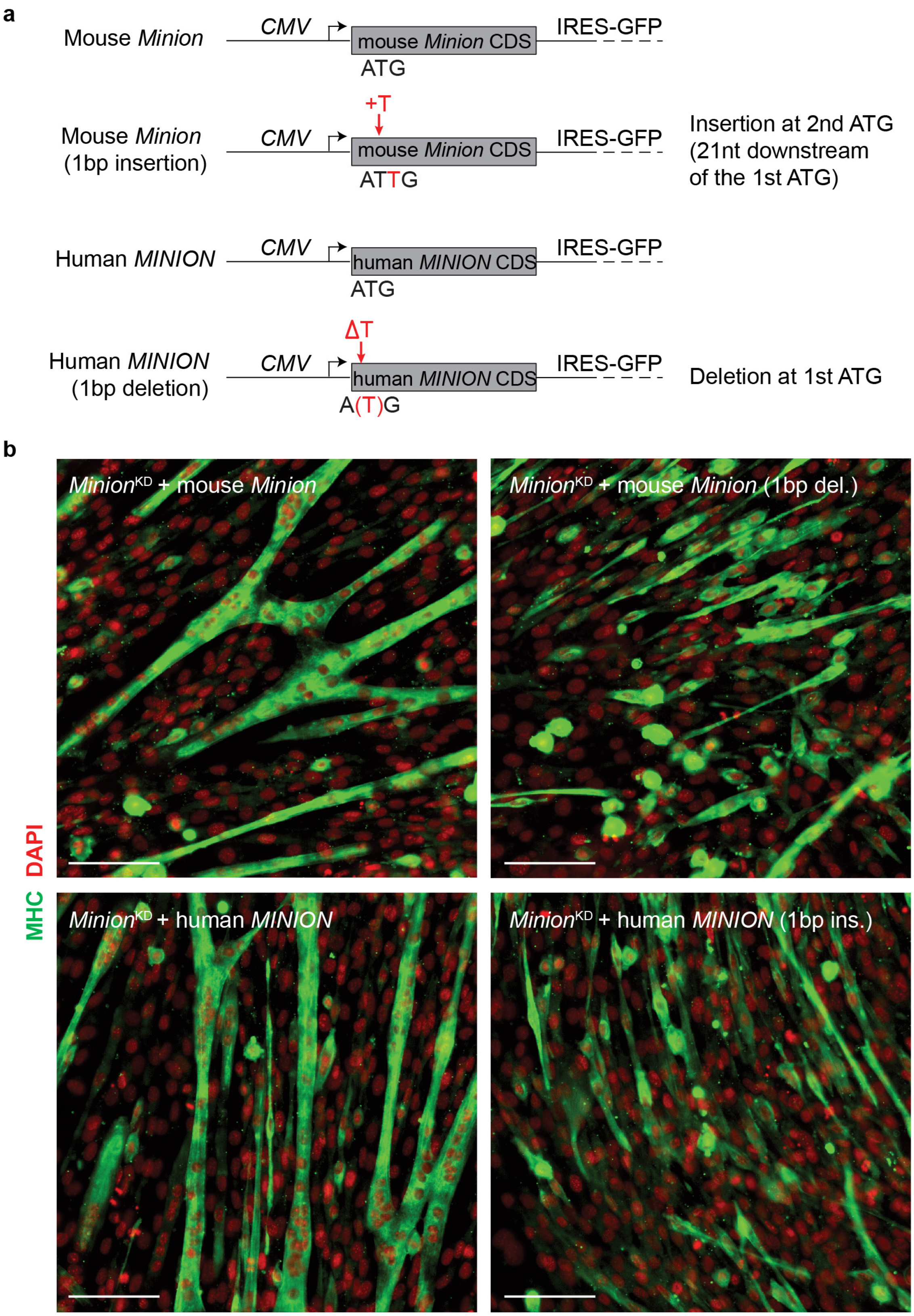

Supplementary Fig. 15. Mouse and human *Minion* transcripts do not function as lncRNAs. (a) Schematic of four retroviral vectors containing either intact mouse/human

Minion CDS, or those with 1bp frameshift (FS) mutations within the start codons (indicated in red). These single base pair mutations are predicted to disrupt the expression of full-length mouse/human Minion proteins without significantly altering RNA sequence. **(b)** Immunofluorescence images of *Minion*^{KD} C2C12 cells with exogenous expression of constructs indicated in (a), and following 5 days in differentiation medium. MHC (green) and DAPI (red) staining are shown. n=2 (6 technical replicates each). Scale bars: 100 μm.

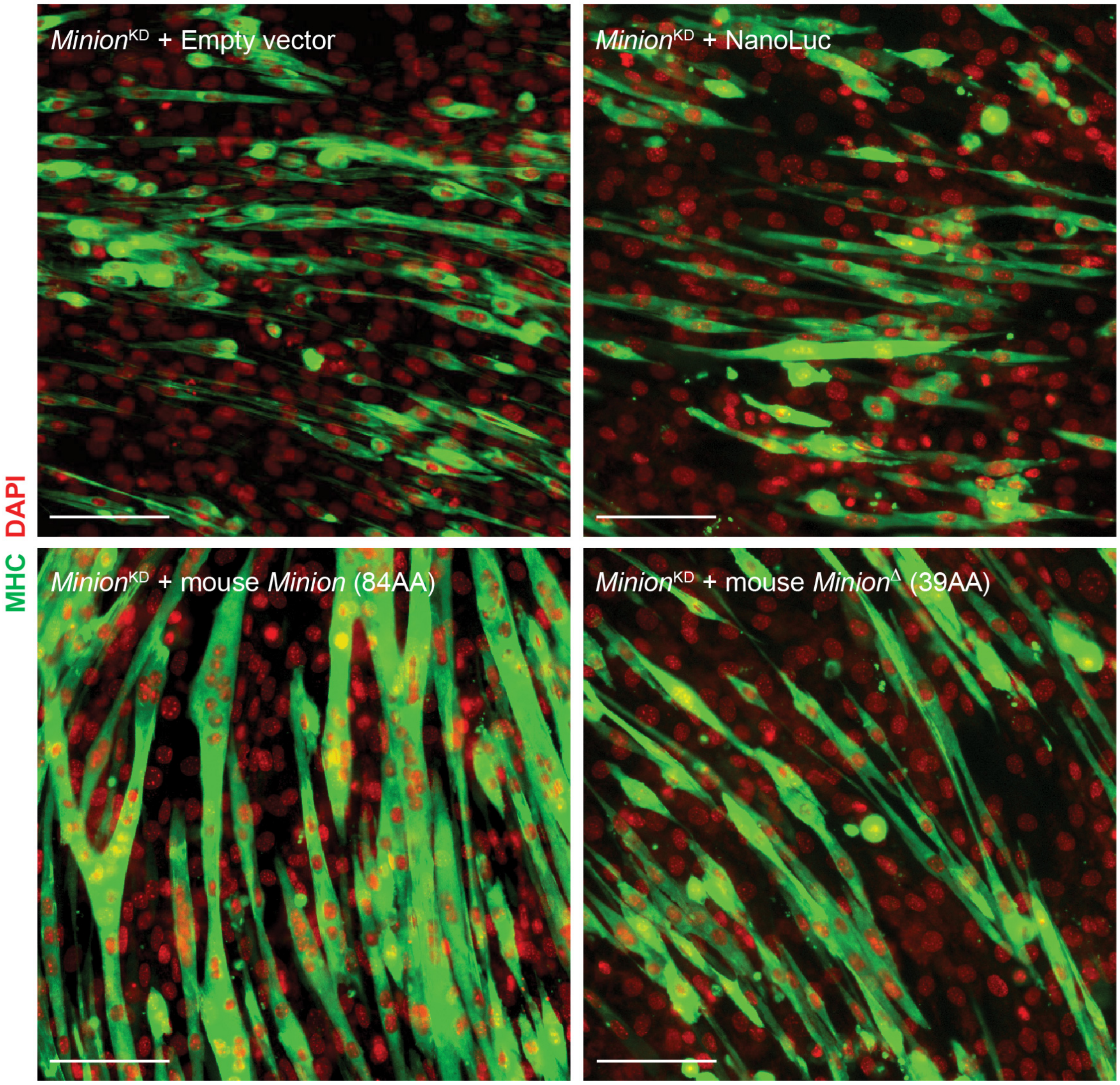

Supplementary Fig. 16. The 39 aa Minion$^{\Delta}$ truncation mutant fails to rescue the *Minion*^{KD} C2C12 fusion defect. Immunofluorescence images of *Minion*^{KD} C2C12 cells transduced with retroviral constructs encoding either empty vector (negative control), NanoLuc (negative control), full-length mouse Minion, or the truncated 39 aa Minion mutant form (predicted from the *Minion*$^{\Delta}$ knockout allele containing the 135bp in-frame deletion). Cells were cultured in differentiation medium for 5 days. MHC (green) and DAPI (red) staining are shown. n=2 (8 technical replicates each). Scale bars: 100 µm.

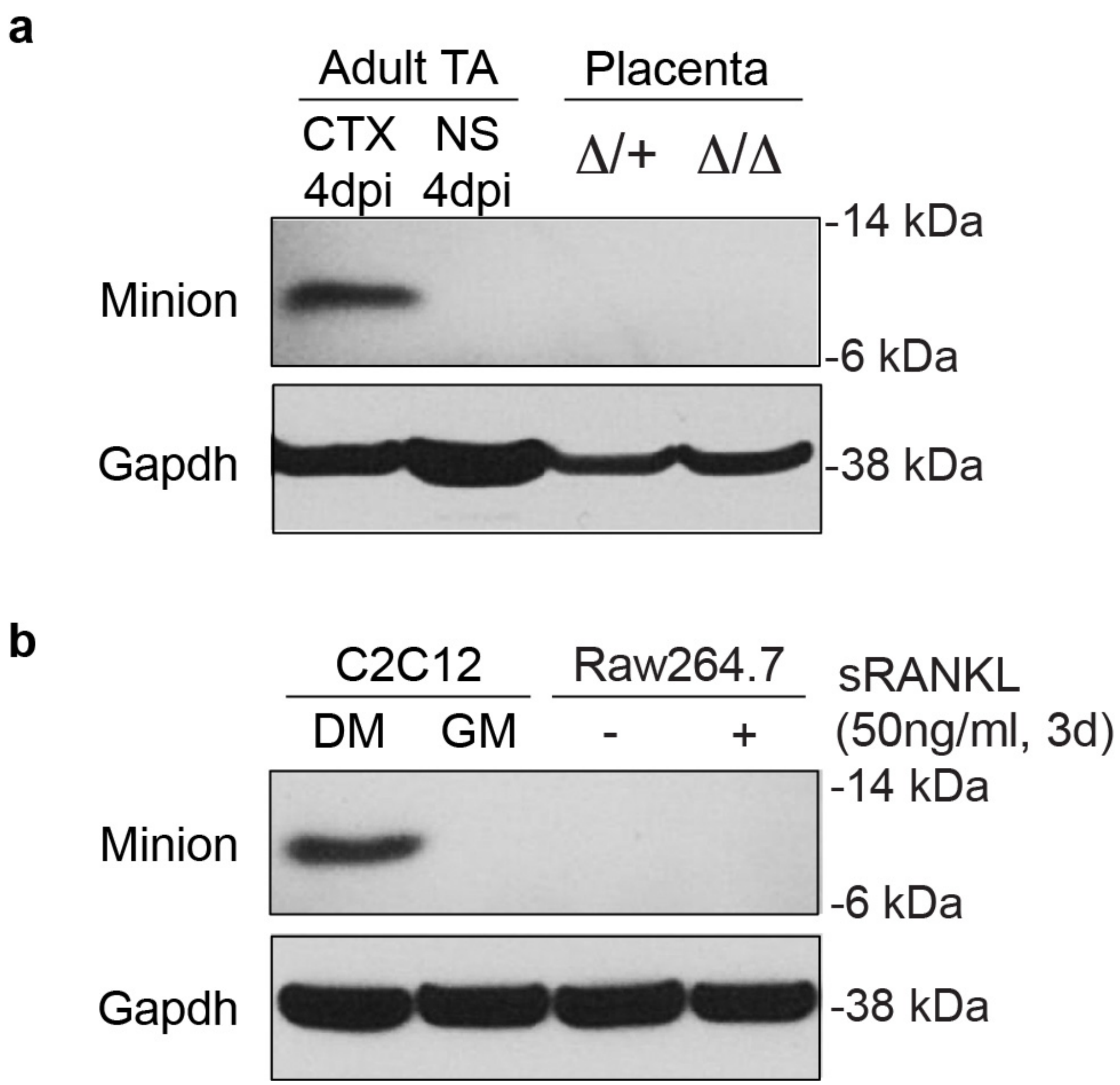

Supplementary Fig. 17. Minion is undetectable in two models of non-myogenic cell-cell fusion. (a) Western blot analysis of Minion expression in placenta. Placenta and periplacental tissue from *Minion*$^{\Delta/+}$ and *Minion*$^{\Delta/\Delta}$ embryos were examined. TA muscles with normal saline injection (NS; day 4) and cardiotoxin injection (CTX; day 4) were used as negative and positive controls, respectively. **(b)** Western blot analysis of Minion expression upon soluble RANKL ligand-induced cell-cell fusion and osteoclast formation in the macrophage line Raw264.7. n=2. C2C12 cells cultured either in growth medium (GM), or in differentiation medium for 5 days (DM) were used as positive and negative controls respectively.

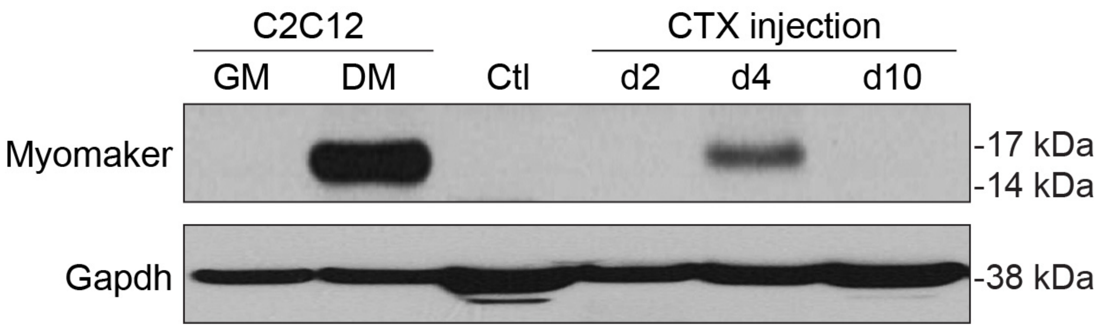

Supplementary Fig. 18. An anti-human Myomaker antibody recognizes endogenous mouse Myomaker. Western blot analysis of C2C12 myoblasts cultured under either growth conditions (GM), or in differentiation conditions for 3 days (DM); and of uninjured (Ctl) or cardiotoxin injured and regenerating TA muscle at different time points (day 2, 4, 10). Antibody incubations were performed as described in Methods. n=3.

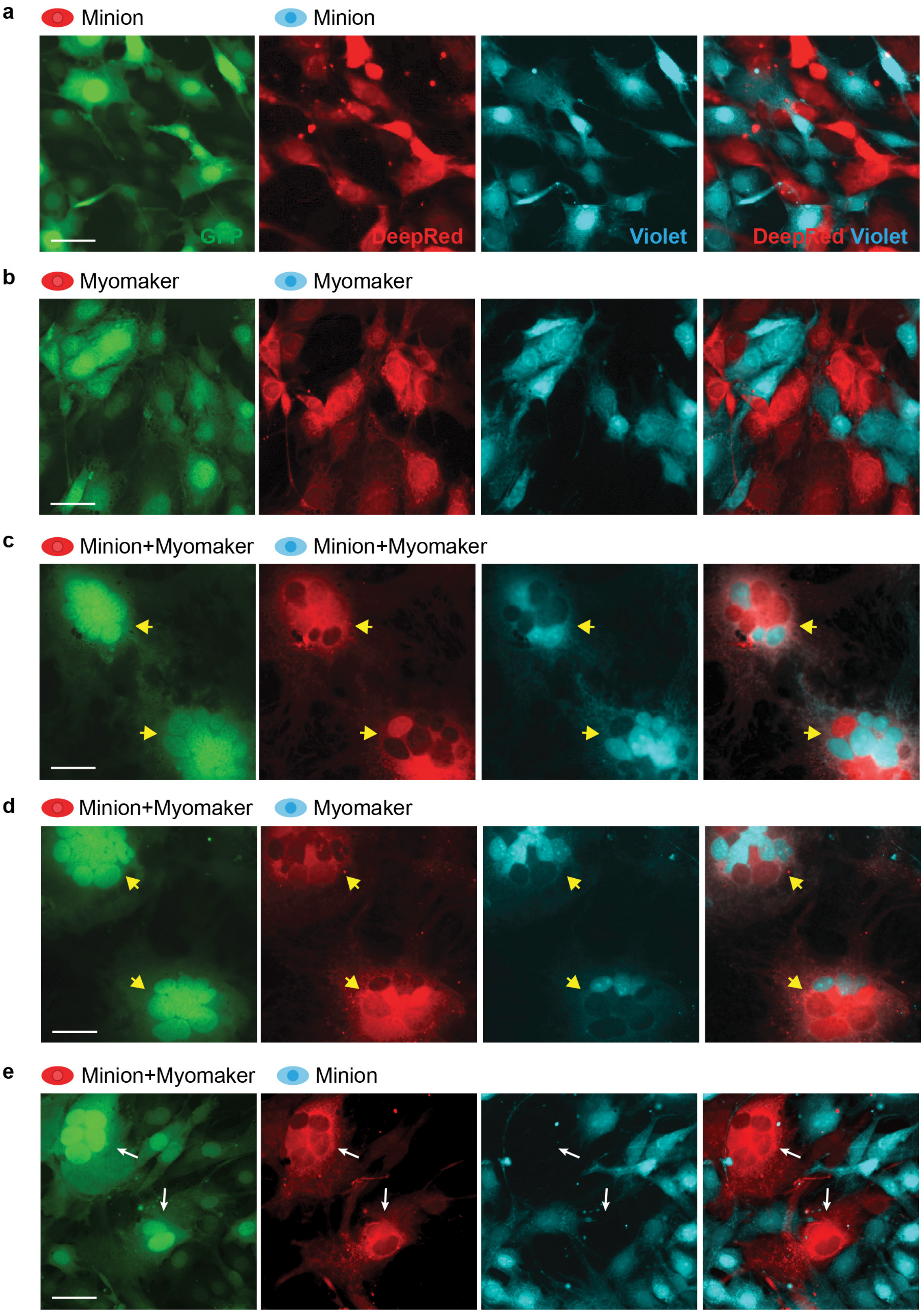

Supplementary Fig. 19. Minion and Myomaker are together sufficient to induce cell-cell fusion, with Minion required only in one cell. Split-channel fluorescence images

are provided of data included in Fig. 7d. Fluorescence images are shown from cell mixing experiments using fibroblasts expressing the indicated combinations of proteins and labeled with either CellTrace Violet (cyan) and CellTracker Deep Red (red) dyes. 10T1/2 fibroblasts were serially infected with retroviruses encoding either Minion, Myomaker or control vectors (omitted in the labeling for simplicity). All vectors contain IRES-GFP downstream of the gene of interest, causing infected cells to express GFP. Relevant cell color and proteins expressed are indicated above each image. Yellow arrowheads indicate syncytia derived from Deep Red$^+$ cells co-expressing Minion and Myomaker that also contain Violet$^+$ nuclei from the second cell type expressing either Myomaker only or Minion and Myomaker together. White arrows indicate syncytia derived from Deep Red$^+$ cells co-expressing Minion and Myomaker that do not contain Violet$^+$ nuclei from the second cell type expressing Minion only. Scale bars: 50μm.

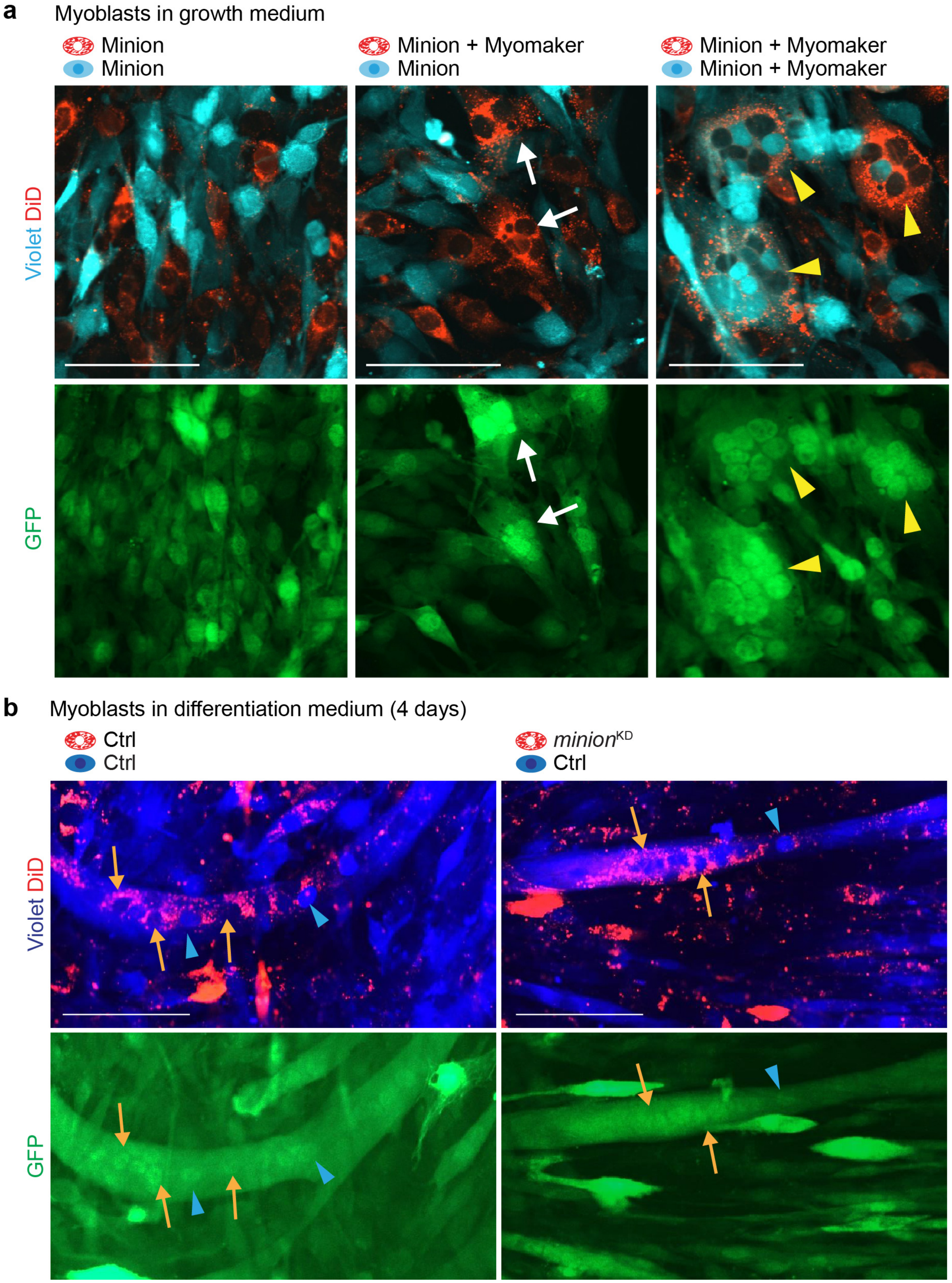

Supplementary Fig. 20. Myoblasts also demonstrate asymmetry between the fusion pair. (a) Fluorescence images from cell mixing experiments using undifferentiated myoblasts in GM expressing the indicated combinations of proteins and labeled with either CellTrace Violet (cyan) and Vybrant DiD (red) dyes. *Minion*^{KD} myoblasts were

serially infected with retroviruses encoding Minion, followed by Myomaker or control vectors (labels omitted for simplicity). All vectors contain IRES-GFP downstream of the gene of interest. Relevant cell color and proteins expressed are indicated above each image. Yellow arrowheads indicate syncytia positive for two dyes and expressing both Minion and Myomaker. White arrows indicate syncytia derived from DiD^{+} cells co-expressing Minion and Myomaker that do not contain $Violet^{+}$ nuclei from the second cell type expressing Minion only. n=2 (8 technical replicates each). **(b)** Fluorescence images from cell mixing experiments using Ctrl and *Minion*^{KD} myoblasts labeled with either CellTrace Violet (blue) and Vybrant DiD (red) dyes. All cellsare GFP positive. The mixed cells were cultured in DM for 4 days. Orange arrows point to DiD-labeled cell derivatives in the fused multinuclear myotubes, cyan arrowheads point to Violet-labeled cell derivatives. n=2 (8 technical replicates each). Scale bars: 100μm (a,b).

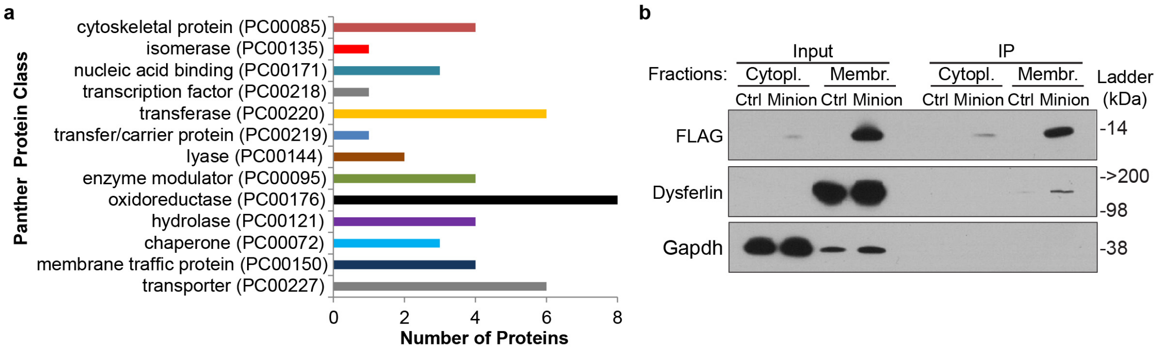

Supplementary Fig. 21. Identification of Minion-interacting proteins. (a) Minion-associated proteins were identified by affinity purification-MS analysis from day 3.5 differentiating C2C12 myoblasts expressing FLAG-tagged Minion (see Supplementary Table 1), and were grouped into protein classes using Panther[4]. The number of significantly enriched proteins in each class is indicated. n=3. **(b)** Western blot confirmation of an example hit. Minion and Gapdh serve as positive and negative controls respectively.

Fig.1b Uncropped Western blots

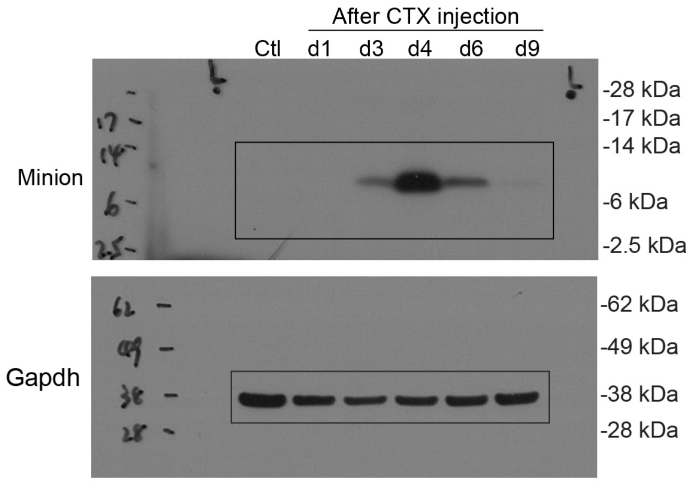

Fig.1c Uncropped Western blots

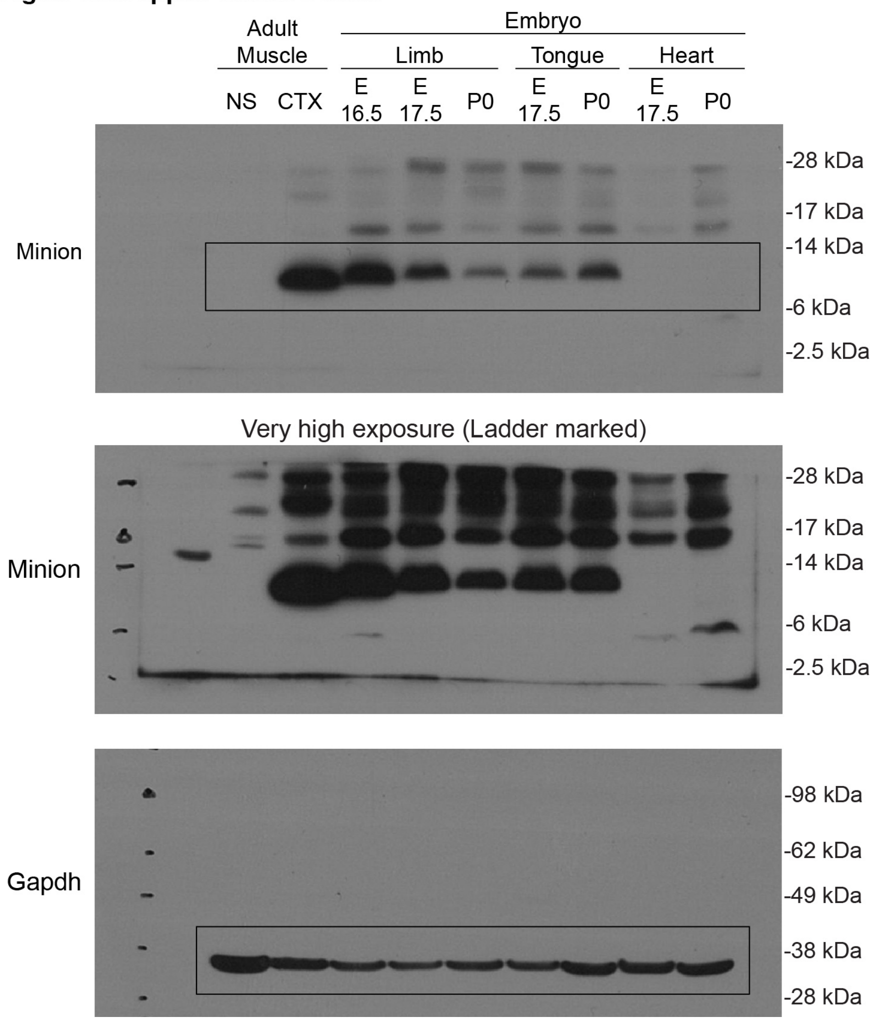

Supplementary Fig. 22. Uncropped Western blot images for Fig. 1b and Fig. 1c.

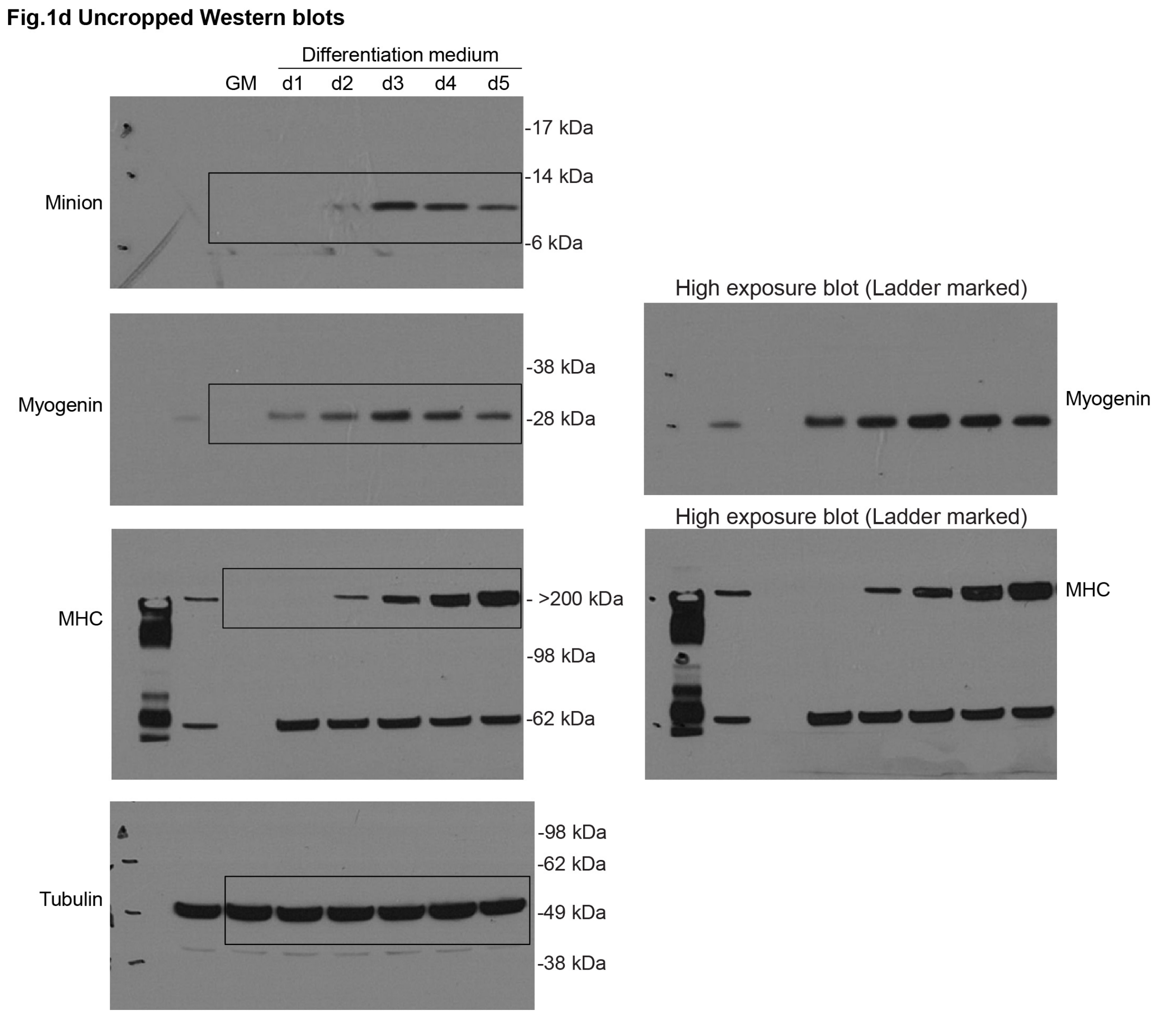

Supplementary Fig. 23. Uncropped Western blot images for Fig. 1d.

Fig.5c Uncropped Western Blots

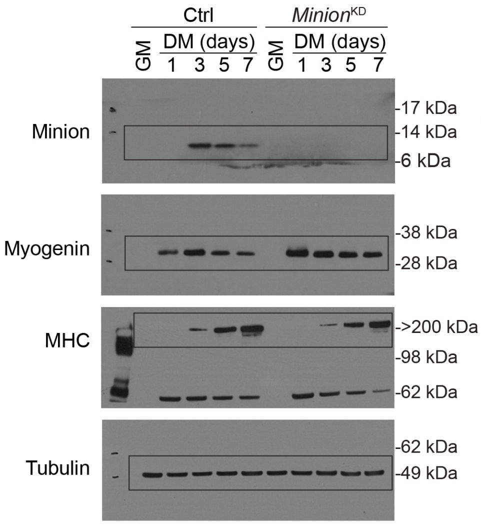

Fig.6c Uncropped Western Blots

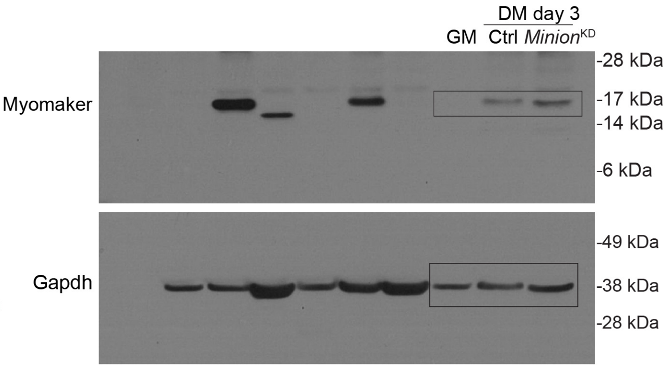

Fig.6e Uncropped Western Blots

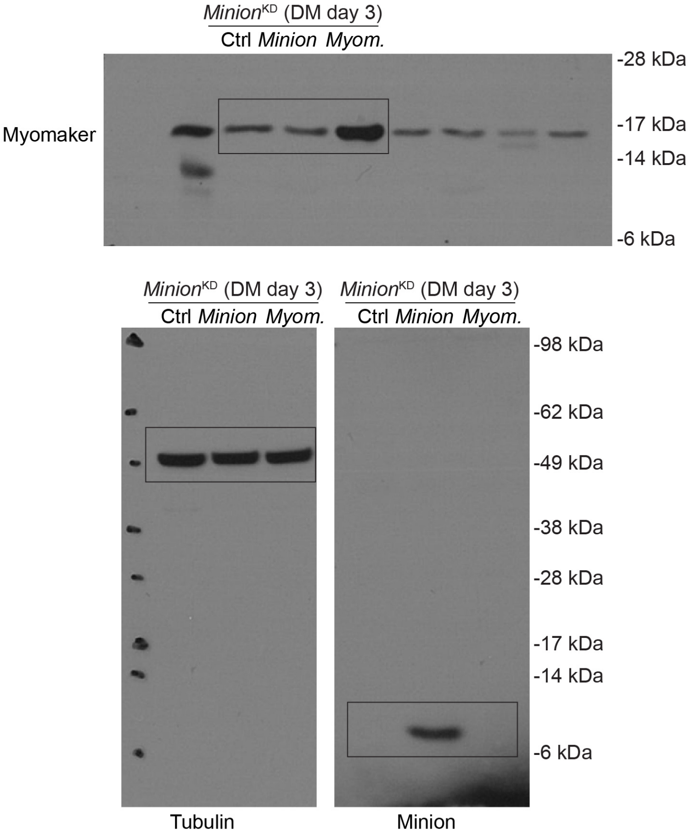

Supplementary Fig. 24. Uncropped Western blot images for Fig. 5c, Fig. 6c and Fig. 6e.

Fig.7a Uncropped Western blots

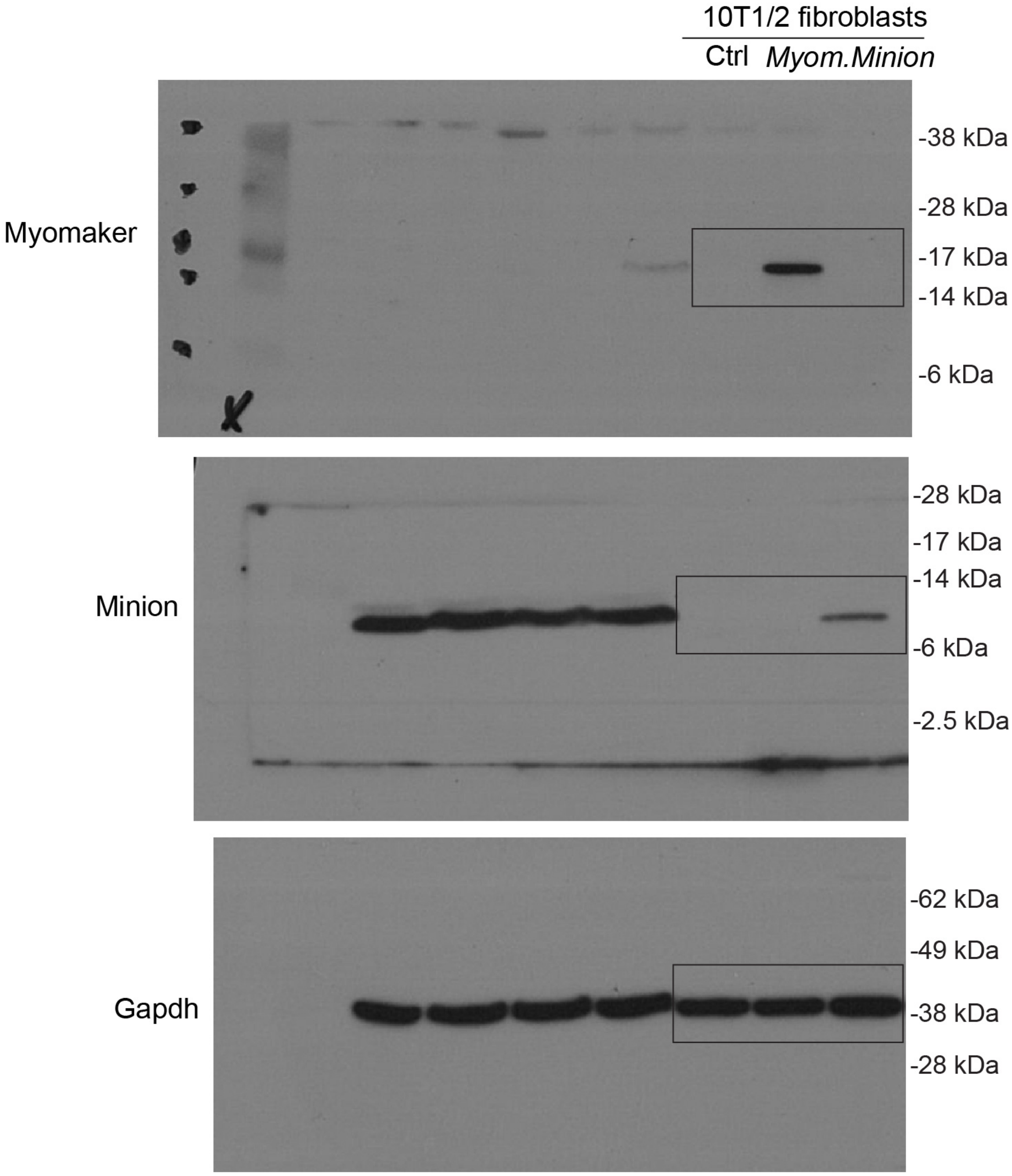

Supplementary Fig. 25. Uncropped Western blot images for Fig. 7a.

a Gating strategy for Ctrl myoblasts

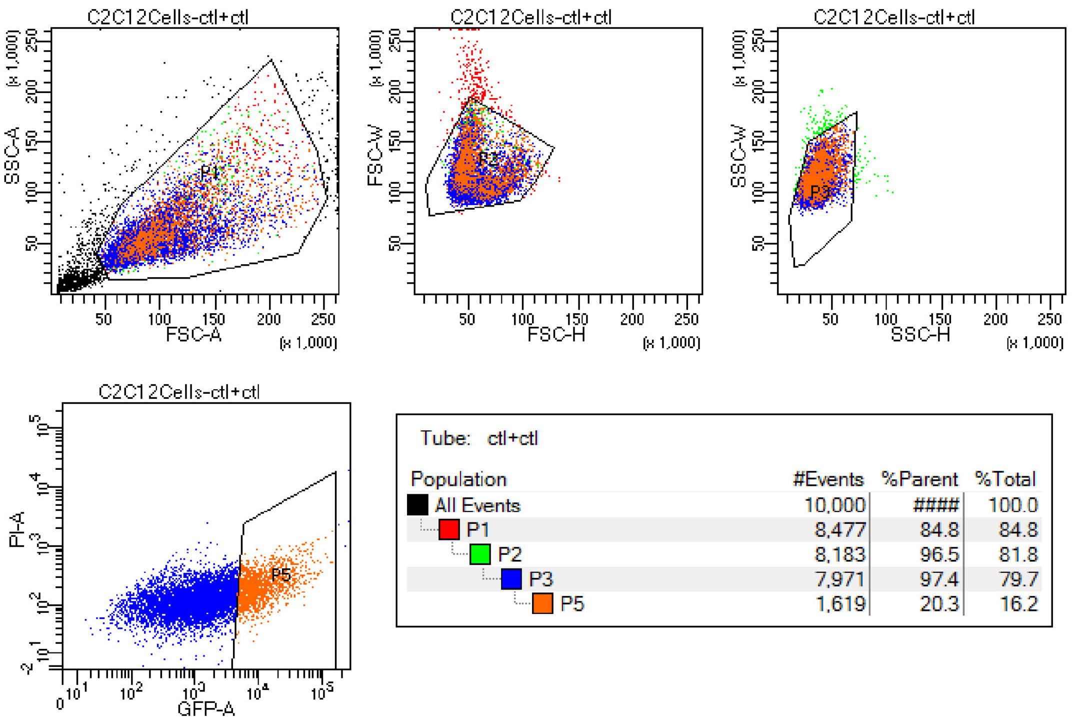

Tube: ctl+ctl

Population	#Events	%Parent	%Total
All Events	10,000	####	100.0
P1	8,477	84.8	84.8
P2	8,183	96.5	81.8
P3	7,971	97.4	79.7
P5	1,619	20.3	16.2

b Gating strategy for *Minion*^KD myoblasts

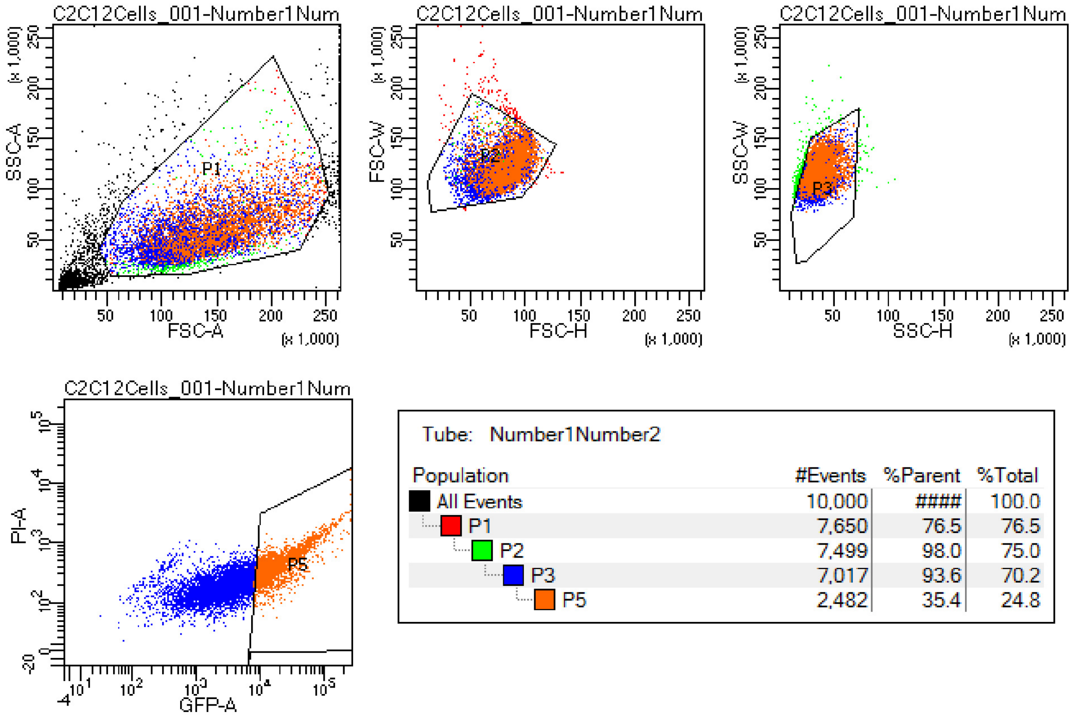

Tube: Number1Number2

Population	#Events	%Parent	%Total
All Events	10,000	####	100.0
P1	7,650	76.5	76.5
P2	7,499	98.0	75.0
P3	7,017	93.6	70.2
P5	2,482	35.4	24.8

Supplementary Fig. 26. Gating strategy for identification and sorting of Ctrl and *Minion*^{KD} myoblasts.

Supplementary Table 1. Proteins significantly enriched in complex with Minion following affinity purification-mass spectrometry.

Description	Accession Number	Molecular Weight	Fisher's Exact Test (p-value) *(p<0.00414)	Number of Spectra	Unique Peptide	Sequence Coverage (%)
Embryonic stem cell-and germ cell-specific protein ESGP OS=Mus musculus GN=Gm7325 PE=2 SV=1	Q2Q5T5	9.6 kDa	< 0.00010	33	3	50
Dysferlin OS=Mus musculus GN=Dysf PE=1 SV=3	DYSF_MOUSE	238 kDa	< 0.00010	23	17	13
Aldehyde dehydrogenase family 3 member B1 OS=Mus musculus GN=Aldh3b1 PE=2 SV=1	AL3B1_MOUSE	52 kDa	< 0.00010	58	15	46
ATPase family AAA domain-containing protein 1 OS=Mus musculus GN=Atad1 PE=1 SV=1	ATAD1_MOUSE	41 kDa	<0.00010	15	7	30
Signal recognition particle 68 kDa protein OS=Mus musculus GN=Srp68 PE=2 SV=2	SRP68_MOUSE	71 kDa	< 0.00010	19	11	25
Calsequestrin-2 OS=Mus musculus GN=Casq2 PE=2 SV=3	CASQ2_MOUSE	48 kDa	< 0.00010	35	9	24
Calcium uniporter protein, mitochondrial OS=Mus musculus GN=Mcu PE=2 SV=2	MCU_MOUSE	40 kDa	< 0.00010	34	12	38
Inositol 1,4,5-trisphosphate receptor type 3 OS=Mus musculus GN=Itpr3 PE=1 SV=3	ITPR3_MOUSE	304 kDa	0.0027	14	11	6
Synaptopodin 2-like protein OS=Mus musculus GN=Synpo2l PE=2 SV=1	SYP2L_MOUSE	103 kDa	0.0024	9	5	8
DnaJ homolog subfamily C member 16 OS=Mus musculus GN=Dnajc16 PE=1 SV=2	DJC16_MOUSE	89 kDa	0.00032	12	9	19
Syntaxin-8 OS=Mus musculus GN=Stx8 PE=1 SV=1	STX8_MOUSE	27 kDa	0.00062	11	4	19
NADH-ubiquinone oxidoreductase 75 kDa subunit, mitochondrial OS=Mus musculus GN=Ndufs1 PE=1 SV=2	NDUS1_MOUSE	80 kDa	0.00010	19	9	20

Adenylate kinase isoenzyme 4, mitochondrial OS=Mus musculus GN=Ak4 PE=2 SV=1	KAD4_MOUSE	25 kDa	0.00010	15	6	52
Sorbin and SH3 domain-containing protein 2 OS=Mus musculus GN=Sorbs2 PE=1 SV=2	SRBS2_MOUSE	132 kDa	0.0024	9	6	7
Vacuolar protein sorting-associated protein 45 OS=Mus musculus GN=Vps45 PE=1 SV=1	VPS45_MOUSE	65 kDa	0.0012	10	7	15
NADH dehydrogenase [ubiquinone] flavoprotein 1, mitochondrial OS=Mus musculus GN=Ndufv1 PE=1 SV=1	NDUV1_MOUSE	51 kDa	0.0022	12	7	24
B-cell receptor-associated protein 31 OS=Mus musculus GN=Bcap31 PE=1 SV=3	BAP31_MOUSE	28 kDa	0.00032	12	7	27
NADH dehydrogenase [ubiquinone] 1 alpha subcomplex subunit 10, mitochondrial OS=Mus musculus GN=Ndufa10 PE=1 SV=1	NDUAA_MOUSE	41 kDa	0.0040	12	7	24
Cluster of Guanine nucleotide-binding protein G(s) subunit alpha isoforms short OS=Mus musculus GN=Gnas PE=1 SV=1 (GNAS2_MOUSE)	GNAS2_MOUSE [7]	46 kDa	0.00012	22	9	34
Acetolactate synthase-like protein OS=Mus musculus GN=Ilvbl PE=2 SV=1	ILVBL_MOUSE	68 kDa	0.0011	22	10	32
Cytoskeleton-associated protein 5 OS=Mus musculus GN=Ckap5 PE=2 SV=1	CKAP5_MOUSE	226 kDa	0.0038	23	13	8
Voltage-dependent anion-selective channel protein 1 OS=Mus musculus GN=Vdac1 PE=1 SV=3	VDAC1_MOUSE	32 kDa	0.00094	28	10	15
Apolipoprotein O-like OS=Mus musculus GN=Apool PE=2 SV=1	APOOL_MOUSE	29 kDa	0.0039	28	9	49
Junctional sarcoplasmic reticulum protein 1 OS=Mus musculus GN=Jsrp1 PE=1 SV=2	JSPR1_MOUSE	36 kDa	0.0023	31	8	53
Tripartite motif-containing protein 72 OS=Mus	TRI72_MOUSE	53 kDa	0.00048	40	16	55

musculus GN=Trim72 PE=1 SV=1						
Cytochrome b-c1 complex subunit 1, mitochondrial OS=Mus musculus GN=Uqcrc1 PE=1 SV=2	QCR1_MOUSE	53 kDa	0.0010	38	13	41
Eukaryotic initiation factor 4A-II OS=Mus musculus GN=Eif4a2 PE=2 SV=2	IF4A2_MOUSE	46 kDa	0.0023	45	9	53
T-complex protein 1 subunit theta OS=Mus musculus GN=Cct8 PE=1 SV=3	TCPQ_MOUSE	60 kDa	< 0.00010	57	22	48
Isovaleryl-CoA dehydrogenase, mitochondrial OS=Mus musculus GN=Ivd PE=1 SV=1	IVD_MOUSE	46 kDa	0.0012	50	16	49
T-complex protein 1 subunit beta OS=Mus musculus GN=Cct2 PE=1 SV=4	TCPB_MOUSE	57 kDa	0.00076	53	20	55
Nascent polypeptide-associated complex subunit alpha, muscle-specific form OS=Mus musculus GN=Naca PE=1 SV=2	NACAM_MOUSE	220 kDa	0.0031	50	23	18
Coproporphyrinogen-III oxidase, mitochondrial OS=Mus musculus GN=Cpox PE=1 SV=2	HEM6_MOUSE	50 kDa	0.0014	57	19	52
T-complex protein 1 subunit alpha OS=Mus musculus GN=Tcp1 PE=1 SV=3	TCPA_MOUSE	60 kDa	0.0035	65	20	52
Very long-chain specific acyl-CoA dehydrogenase, mitochondrial OS=Mus musculus GN=Acadvl PE=1 SV=3	ACADV_MOUSE	71 kDa	0.0017	72	26	57
Trifunctional enzyme subunit beta, mitochondrial OS=Mus musculus GN=Hadhb PE=1 SV=1	ECHB_MOUSE	51 kDa	< 0.00010	93	22	68
Trifunctional enzyme subunit alpha, mitochondrial OS=Mus musculus GN=Hadha PE=1 SV=1	ECHA_MOUSE	83 kDa	< 0.00010	201	33	66
ATP synthase subunit beta, mitochondrial OS=Mus musculus	ATPB_MOUSE	56 kDa	< 0.00010	231	26	77

GN=Atp5b PE=1 SV=2						
Cytoplasmic dynein 1 heavy chain 1 OS=Mus musculus GN=Dync1h1 PE=1 SV=2	DYHC1_MOUSE	532 kDa	< 0.00010	444	157	46
Cluster of Myc box-dependent-interacting protein 1 OS=Mus musculus GN=Bin1 PE=1 SV=1 (BIN1_MOUSE)	BIN1_MOUSE [3]	64 kDa	0.003	75	16	42

Supplementary Table 2. List of sequences of primers and gRNA protospacers mentioned in the Methods section

**Name**	**Sequence**
gRNA 1 protospacer	GGACCGGGCCGTCGTGGAGG
gRNA 2 protospacer	CCAGAGTGGACCACTCCCAG
gRNA 3 protospacer	GGCGGGCAACAGGCAGCAGC
gRNA 4 protospacer	CTGGGAGTGGTCCACTCTGG
gRNA oligonucleotide 1	TTAATACGACTCACTATAG-(gRNA protospacer)-GTTTTAGAGCTAGAAATAGCAAGTTAAAATAAGGCTAGTCG
gRNA oligonucleotide 2	AAAAAGCACCGACTCGGTGCCACTTTTTCAAGTTGATAACGGACTAGCCTTATTTTAACTT
Seq-F (genotyping)	GAGTGAACTCCTTAACCAGCTTTC
Seq-R (genotyping)	GCGTTGCTGTTTCCAGGACCCGTG
Fwd (genotyping)	CAAAGGGAGGGAGGGATTAAAG
Rev (genotyping)	CAGAGAGGAAGGGTCAATCAAC
Fwd-2 (genotyping)	AACACAATCTGTAGCCTGCTAGGAG
Rev-2 (genotyping)	TATAAGCTGAAGGGAGGACTCCAC
Minion-F (RT-PCR)	GGACCACTCCCAGAGGAAGGA
Minion-R (RT-PCR)	GGACCGACGCCTGGACTAAC
Gapdh -F (RT-PCR)	AGGTCGGTGTGAACGGATTTG
Gapdh -R (RT-PCR)	TGTAGACCATGTAGTTGAGGT

Supplementary Table 3. Antibody and ECL substrate information for Western blots

Primary antibody	Dilution	Primary antibody incubation conditions	HRP conjugated secondary antibody	Dilution	Secondary antibody incubation conditions	ECL substrate used most	Protein size detected
Sheep anti-Gm7325/Minion (R&D AF4580)	1:1000 (1ug/ml)	4 °C overnight	Donkey anti-sheep (Thermo Fisher A16047)	1:5000	Room temperature (RT) for 1-2 hours	Luminata Forte (EMD Millipore WBLUF0100; increased sensitivity). Diluted with equal volume of water.	10-12kDa
Mouse anti-Gapdh (EMD Millipore MAB374)	1:20,000	RT 30 min-1 hour	Goat anti-mouse (Thermo Fisher A16078)	1:20,000	RT 1 hour	SuperSignal West Pico (Thermo Fisher 34080)	36kDa
Mouse anti-alpha-Tubulin (Sigma T9026)	1:20,000	RT 1-2 hours	Same as above	1:20,000	RT 1-2 hours	SuperSignal West Pico	50kDa
Mouse anti- MHC MY32 (Sigma M4276)	1:1000	4 °C overnight	Same as above	1:5000	RT 1-2 hours	Luminata Forte	>200kDa
Mouse anti-Myogenin (DSHB F5Dc)	1:200	4 °C overnight	Same as above	1:5000	RT 1-2 hours	Luminata Forte	25kDa
Mouse anti-Desmin clone D33 (DAKO M0760)	1:200	4 °C overnight	Same as above	1:5000	RT 1-2 hours	Luminata Forte	53kDa
Mouse anti-Dysferlin (Thermo fisher MA5-12568)	1:150	4°C overnight	Same as above	1:5000	RT 1-2 hours	SuperSignal West Pico	240kDa
Rabbit anti-MyoD1 (Novus Biologicals NBP1-54153)	1:5000	4 °C overnight	Goat anti-rabbit (Cell Signaling #7074)	1:2000	RT 1-2 hours	Luminata Forte	34kDa
Rabbit anti-N-Cadherin(Cell signaling #4061)	1:1000	4 °C overnight	Same as above	1:2000	RT 1-2 hours	Luminata Forte	100kDa
Rabbit anti-Calnexin (Novus Biologicals NB100-1965SS)	1:1000	4 °C overnight	Same as above	1:2000	RT 1-2 hours	SuperSignal West Pico	67kDa
Rabbit anti-H2B (Cell signaling #8135)	1:1000	4 °C overnight	Same as above	1:2000	RT 1-2 hours	Luminata Forte	14kDa
Rabbit anti-Vimentin [EPR3776] (Abcam ab92547)	1:10,000	4°C overnight	Same as above	1:2000	RT 1-2 hours	SuperSignal West Pico	54kDa

Rabbit anti-pan TGFbeta antibody(Cell signaling #3711)	1:1000	4°C overnight	Same as above	1:2000	RT 1-2 hours	SuperSignal West Pico	12kDa (mature, secrected)
Rabbit anti-Human TMEM8C (Myomaker) (Abcam ab188300)	1:200	4 °C at least two days	Same as above	1:800	4 °C overnight plus RT 3 hours; or 4 °C two days	Luminata Forte	Appear as around 17kDa; estimated as 25kDa.
HRP conjugated mouse anti-FLAG M2 (Sigma A8592)	1:10,000	4 °C overnight	--	--	--	SuperSignal West Pico	

Supplementary Table 4. shRNA target sequence and oligonucleotide design information

shRNA construct name	Target sequence	Oligonucleotide names (S: Sense; A: Antisense)	Oligonucleotide sequence
m*Minion*-U1	GCTAAGAGGTGGTATTTAA	3'UTR shRNA_1S	TTTGCTAAGAGGTGGTATTTAATTCAAGAGATTAAATACCACCTCTTAGCTTTTT
		3'UTR shRNA_1A	CTAGAAAAAGCTAAGAGGTGGTATTTAATCTCTTGAATTAAATACCACCTCTTAG
m*Minion*-U2	GCAGCAGGTAGTCAATAAACG	3'UTR shRNA_2S	TTTGCAGCAGGTAGTCAATAAACGTTCAAGAGACGTTTATTGACTACCTGCTGCTTTTT
		3'UTR shRNA_2A	CTAGAAAAAGCAGCAGGTAGTCAATAAACGTCTCTTGAACGTTTATTGACTACCTGCTG
m*Minion*-C1	GCTGTCTGCTCTTTGTCCT	CDS shRNA_1S	TTTGCTGTCTGCTCTTTGTCCTTTCAAGAGAAGGACAAAGAGCAGACAGCTTTTT
		CDS shRNA_1A	CTAGAAAAAGCTGTCTGCTCTTTGTCCTTCTCTTGAAAGGACAAAGAGCAGACAG
m*Minion*-C2	GTGGACCACTCCCAGAGGA	CDS shRNA_2S	TTTGTGGACCACTCCCAGAGGATTCAAGAGATCCTCTGGGAGTGGTCCACTTTTT
		CDS shRNA_2A	CTAGAAAAAGTGGACCACTCCCAGAGGATCTCTTGAATCCTCTGGGAGTGGTCCA
Control (Ctrl)	GACGAACACTTCTTCATCG	Ctrl shGL3_S	TTTGACGAACACTTCTTCATCGTTCAAGAGACGATGAAGAAGTGTTCGTCTTTTT
		Ctrl shGL3_A	TAGAAAAAGACGAACACTTCTTCATCGTCTCTTGAACGATGAAGAAGTGTTCGT

References

1 Mohun, T. *et al.* Deciphering the Mechanisms of Developmental Disorders (DMDD): a new programme for phenotyping embryonic lethal mice. *Disease models & mechanisms* **6**, 562-566, doi:10.1242/dmm.011957 (2013).

2 Petryszak, R. *et al.* Expression Atlas update--a database of gene and transcript expression from microarray- and sequencing-based functional genomics experiments. *Nucleic acids research* **42**, D926-932, doi:10.1093/nar/gkt1270 (2014).

3 Diez-Roux, G. *et al.* A high-resolution anatomical atlas of the transcriptome in the mouse embryo. *PLoS biology* **9**, e1000582, doi:10.1371/journal.pbio.1000582 (2011).

4 Mi, H. *et al.* PANTHER version 11: expanded annotation data from Gene Ontology and Reactome pathways, and data analysis tool enhancements. *Nucleic acids research* **45**, D183-D189, doi:10.1093/nar/gkw1138 (2017).
